# Mechanism of *Pantoea ananatis* in the biocontrol of rice bacterial leaf blight

**DOI:** 10.3389/fmicb.2026.1722838

**Published:** 2026-02-04

**Authors:** Ye Tian, Wenting Lei, Jiayi Zhang, Ying Shen, Jianfei Lu, Munazza Ijaz, Alhassan Alrafaie, Temoor Ahmed, Chengqi Yan, Bin Li

**Affiliations:** 1 State Key Laboratory of Rice Biology and Breeding, Ministry of Agriculture and Rural Affairs Key Laboratory of Molecular Biology of Crop Pathogens and Insect Pests, Hangzhou, China; 2 Zhejiang Key Laboratory of Biology and Ecological Regulation of Crop Pathogens and Insects, Hangzhou, China; 3 Zhejiang Engineering Research Center for Biological Control of Crop Pathogens and Insect Pests, Zhejiang Provincial Key Laboratory of Agricultural Microbiomics, Institute of Biotechnology, Zhejiang University, Hangzhou, China; 4 Station for the Plant Protection and Quarantine and Control of Agrochemicals of Zhejiang Province, Hangzhou, China; 5 Department of Medical Laboratory, College of Applied Medical Sciences in Al-Kharj, Prince Sattam Bin Abdulaziz University, Al-Kharj, Saudi Arabia; 6 Xianghu Laboratory, Hangzhou, China; 7 Department of Plant Biotechnology, Korea University, Seoul, South Korea; 8 Modern Agriculture College, Zhejiang Wanli University, Ningbo, China

**Keywords:** biological control, microbial community, nutrient competition, *Pantoea ananatis*, rice bacterial leaf blight, *Xanthomonas oryzae* pv. *oryzae*

## Abstract

**Introduction:**

Rice bacterial leaf blight, caused by *Xanthomonas oryzae* pv. *oryzae* (*Xoo*), is a highly destructive disease. Within the rice-*Xoo* pathosystem, *Pantoea ananatis* exhibits a dual role, functioning both as a pathogen and as a biocontrol agent, underscoring the need to clarify its speciffc functions for effective disease management.

**Methods:**

Isolated strains ZJU1-ZJU18 were identified using multi-locus sequence analysis, core-genome phylogenomic analysis, and average nucleotide identity. The population density of *Xoo* in rice leaves was determined by plate counting and qPCR to evaluate the inhibitory effect of *P. ananatis* on its growth.

**Results and discussion:**

The isolated strains ZJU1-ZJU18 were all identiffed as *P. ananatis*, and they exhibited plant growth-promoting traits, including phosphate solubilization, siderophore production, and indole-3-acetic acid synthesis. Furthermore, strains ZJU1-ZJU18 did not induce rice bacterial leaf blight symptoms under the experimental conditions, with the lesion inhibition rate against this disease ranging from 95.14 to 97.92%. Mechanistic investigations revealed that *P. ananatis* suppressed *Xoo* via nutrient competition, dominating co-culture systems (>90% relative abundance) and reducing *Xoo* colonization on rice leaves by 96.78–99.00%. *Xoo* infection enhanced *P. ananatis* colonization, likely by modifying the leaf microenvironment. Furthermore, the results of species composition analysis showed that *P. ananatis* could alter the structure and diversity of the microbial community in rice leaves and reduce the abundance of *Xanthomonas* species. The principal coordinate analysis indicated that *P. ananatis* had a more signiffcant impact on the microbial community composition than *Xoo*. This study found that *P. ananatis* may inhibit the pathogen *Xoo* through nutrient competition and reshape the microbial structure at the community level.

## 1 Introduction

The bacterium *Xanthomonas oryzae* pv. *oryzae* (*Xoo*) has become a pervasive threat to rice production across diverse agroecological zones globally (Ramprasad et al., 2024). The occurrence and severity of the disease are influenced by rice cultivar., growth stage, geographic location, and environmental conditions (Duku et al., 2016; Chumpol et al., 2022; Sumuni et al., 2024). The pathogen *Xoo* enters through wounds or hydathodes, spreads systemically via xylem vessels, colonizes the mesophyll tissues of rice, and predominantly infects leaves and leaf sheaths (Timilsina et al., 2020; Cao et al., 2021). Due to the spread of *Xoo* through straw, seeds, and water, disease outbreaks are most severe under high humidity, heavy rainfall, and typhoon weather conditions (Gnanamanickam et al., 1999; Ritbamrung et al., 2025).

Although some new methods have been reported to control plant diseases (Ahmed et al., 2023; Noman et al., 2023), biological control represents an environmentally sustainable alternative to chemical control, offering advantages such as ecological safety, adaptability, minimal impact on non-target organisms, food safety, and sustained efficacy over time (Liu et al., 2023; Zhang et al., 2024; He et al., 2025). In the management of rice bacterial leaf blight, biocontrol bacteria have demonstrated promising effects. These bacteria not only suppress *Xoo* growth through the secretion of antibacterial metabolites but also diminish its competitive advantage through mechanisms such as eliciting rice immune responses and competing for nutrients and ecological niches (Ooi et al., 2022; Islam et al., 2023).

*Pantoea* can be isolated from animals, humans, plants, water, and soil, and exists in ecological roles as pathogens, symbiotic bacteria, endophytic bacteria, and saprophytic bacteria, demonstrating a high degree of ecological diversity (Walterson and Stavrinides, 2015; Lv et al., 2022). *Pantoea* shows high genetic similarity among its members (Azizi et al., 2020; Kini et al., 2021). To date, the genus has been classified into over 25 species that share phenotypic similarities, among which *P. ananatis*, *P. agglomerans*, *P. dispersa*, *P. vagans*, and *P. stewartii* have been the most extensively studied (Toh et al., 2019; Arayaskul et al., 2020; Dahiya et al., 2024; Tian et al., 2024).

In plant ecosystems, *Pantoea* species exhibit both beneficial and detrimental effects. Certain *Pantoea* strains can promote crop growth and enhance plant health through mechanisms such as nutrient provision, phytohormone synthesis, suppression of plant pathogens, induction of broad-spectrum plant resistance, and alleviation of abiotic stresses, including heavy metal pollution (Sun et al., 2022; Krishnappa et al., 2024). Conversely, some *Pantoea* strains can cause the plant leaf spot disease, leaf blight disease, and soft rot disease, leading to significant economic losses (Xue et al., 2021; Yu et al., 2022; Luna et al., 2023; Wang et al., 2025).

*P. ananatis* exhibits a complex dualistic role in rice disease systems. Duan et al. (2025) has identified *P. ananatis* JMS78-1 as a potential pathogen responsible for rice bacterial leaf blight. However, Jiang et al. (2025) has reported *P. ananatis* YN26 can be used as a biological control agent to control rice bacterial leaf blight. The role of *P. ananatis* in rice production has attracted considerable interest and has become a hot topic of current research.

In this study, *P. ananatis* strains ZJU1-ZJU18 were isolated from rice leaves affected by bacterial leaf blight. Through *in vitro* and *in vivo* experiments, we investigated the pathogenicity of strains ZJU1-ZJU18 and their inhibitory effects on *Xoo*. Using strain ZJU2 as a model, molecular biology techniques and microbiome analysis were employed to elucidate the mechanism by which *P. ananatis* modulates rice bacterial leaf blight. This study aims to clarify the relationship between *P. ananatis* and disease development, and provide novel strategies for disease management.

## 2 Materials and methods

### 2.1 Bacterial strains, plasmid and reagents

In this study, *P. ananatis* strains ZJU1-ZJU18 were isolated from rice leaves affected by rice bacterial leaf blight, collected from field sites in Wenzhou, Hangzhou, Quzhou, Shaoxing, Ningbo, Taizhou, Jinhua, Suqian, Huai’an, Chizhou, Guiping, and Jiangmen cities. The *Xoo* strains C2, PXO99^A^, and N1, along with the plasmids pBBR1MCS-5, pBBR1MCS-RFP, and pRADK-GFP, were obtained from the Plant Bacteriology Laboratory at Zhejiang University. The plasmid pBBR1MCS2-*lux*CDABE was generously provided by the Plant-Microbe Interaction Laboratory at Zhejiang University.

Bacterial isolation was performed using tryptic soy agar (TSA) medium. *Xoo* and *P. ananatis* were grown in nutrient broth (NB) medium at 30 °C. The composition of media used in this study are described below. TSA medium was prepared with 15 g tryptone, 5 g soybean peptone, 5 g NaCl and 15 g agar per liter of distilled water. The pH was adjusted to 7.0 prior to autoclaving at 121 °C for 15 min. NB medium was prepared with 10 g tryptone, 3 g beef extract, and 5 g NaCl per liter of distilled water. The pH was adjusted to 7.0 prior to autoclaving at 121 °C for 15 min. Methyl Red-Voges Proskauer (MR-VP) medium was prepared with 5 g peptone, 5 g glucose, and 3.8 g K_2_HPO_4_ per liter of distilled water. The pH was adjusted to 7.0, and the medium was sterilized by autoclaving at 121 °C for 15 min. Nitrogen-limited (NL) medium was prepared with 10 g glucose, 1 g KH_2_PO_4_, 1 g K_2_HPO_4_, 0.2 g MgSO_4_·7H_2_O, 0.5 g NaCl, 0.1 g CaSO_4_, 0.05 g FeSO_4_, and 0.05 g MnSO_4_ per liter of distilled water. The pH was adjusted to 7.0, and the medium was sterilized by autoclaving at 121 °C for 15 min. Carbon-limited (CL) medium was prepared with 1 g KH_2_PO_4_, 1 g K_2_HPO_4_, 0.5 g NH_4_Cl, 0.2 g MgSO_4_·7H_2_O, 0.5 g NaCl, 0.1 g CaSO_4_, 0.05 g FeSO_4_, and 0.05 g MnSO_4_ per liter of distilled water. The pH was adjusted to 7.0, and the medium was sterilized by autoclaving at 121 °C for 15 min. Chrome azurol S (CAS) medium was prepared by combining, per liter, 100 mL of 10 × PIPES buffer, 20 g glucose, 10 g peptone, 0.5 g MgSO_4_·7H_2_O, and 0.5 g of CaCl_2_. Following sterilization by autoclaving (121 °C, 15 min) and cooling, 100 mL of sterile, pre-mixed CAS detection solution (containing Chrome Azurol S, FeCl_3_·6H_2_O, and hexadecyltrimethylammonium bromide) was added aseptically.

Protease, cellulase, amylase, Mongina organic/inorganic phosphorus media, CAS medium, oxidase test strips, methyl red (MR) reagent, voges proskauer (VP) reagent, hydrogen peroxide, and Lugol’s iodine solution-were purchased from Hopebio biotechnology Co., Ltd. Both media were prepared in sterile distilled water and adjusted to pH 7.0 at 25 °C. Bacterial DNA from rice leaves was extracted using the QIAGEN PowerSoil Kit. Quantitative PCR (qPCR) assay was performed using HisenBio SYBR Green Master Mix. Besides, kanamycin (Km) and gentamicin (Gen) sulfate were applied at a concentration of 50 μg/mL.

### 2.2 Isolation and identification of *Pantoea ananatis*

#### 2.2.1 Isolation of *Pantoea ananatis*

*Pantoea* strains were isolated following a modified protocol adapted from Yang et al. (2020). Rice leaves were surface-sterilized by wiping with 75% ethanol-soaked cotton, followed by immersion in 1.2% (*v/v*) sodium hypochlorite solution for 5 min and rinsing 3 times with sterile water. The absence of bacterial growth in the final rinse water confirmed effective surface sterilization. The sterilized leaves were homogenized in 1 mL of sterile water containing sterile steel beads using a leaf tissue grinder for 1 min. After brief centrifugation, 100 μL of the supernatant was collected, serially diluted, and spread evenly onto TSA plates. The plates were incubated at 30 °C for 10 d. Individual colonies were picked and purified via repeated streaking on TSA to obtain pure cultures. Isolated single colonies were amplified using 16S-F/R primers, and the resulting PCR products were sequenced bidirectionally. Based on the sequencing results analyzed through the NCBI database, colonies identified as belonging to the genus *Pantoea* were selected for subsequent research.

#### 2.2.2 Construction of *16S rRNA* phylogenetic tree

The method for constructing the *16S rRNA* phylogenetic tree was modified based on Tambong (2019). First, the *16S rRNA* genes were amplified from the isolated *Pantoea* strains, and the resulting PCR products were subjected to bidirectional sequencing. Subsequently, the corresponding *16S rRNA* gene sequences of related *Pantoea* strains were retrieved from the NCBI database and aligned using Clustal W (v2.1) for multiple sequence alignment. Finally, based on the aligned sequences, a phylogenetic tree was constructed using the neighbor-joining (NJ) method in MEGA11 software.

#### 2.2.3 Construction of multi-locus sequence analysis phylogenetic tree

Multi-locus sequence analysis (MLSA) can overcome the limitations of *16S rRNA* gene sequencing in species-level resolution and providing a reliable taxonomic foundation for subsequent research (Komaki, 2022). The method for constructing the MLSA phylogenetic tree was modified based on Tambong (2019). First, the conserved genes *atpD*, *fusA*, *gyrB*, and *rpoB* were amplified from *Pantoea* strains identified by *16S rRNA* analysis, using gene-specific primers (Supplementary Table S1). *Pantoea* strains using amplification primers, and the resulting PCR products were subjected to bidirectional sequencing using sequencing primers. The primers used in this study are listed in Table 1. The NCBI accession numbers of the *atpD*, *fusA*, *gyrB*, and *rpoB* genes for strains ZJU1-ZJU18 are provided in Supplementary Table S2.

**Table 1:** Population sizes of *Xoo* and *P. ananatis* after co-culture in different broth.

Broth	*Xoo* population (CFU/mL)	*P. ananatis* population (CFU/mL)
NB	8.33 × 10^6^	4.33 × 10^9^
NL	1.60 × 10^5^	1.20 × 10^9^
CL	2.00 × 10^6^	1.67 × 10^8^

For a robust phylogenetic analysis, a comprehensive set of reference sequences was obtained from the NCBI database. The selection included type strains and additional representative strains across *Pantoea* species, with an emphasis on groups phylogenetically close to the isolates of this study. The sequencing reads for each gene were separately assembled and verified using SeqMan Pro (DNASTAR v15) to obtain a single consensus sequence for each strain. Subsequently, multiple sequence alignments for each gene set were independently performed using Clustal W (v2.1). After manual inspection of all gene alignments, they were concatenated head-to-tail in the order of *atpD-gyrB-rpoB-fusA* to generate an overall supermatrix for phylogenetic analysis. The concatenated alignment was manually trimmed in MEGA11 to remove non-aligned regions at both ends of all sequences and to delete any alignment columns containing gaps in more than 50% of the sequences, ensuring higher homology of the sites used for tree inference. Finally, phylogenetic trees were constructed in MEGA11 using the NJ and maximum likelihood (ML) methods, respectively. Branch support for the trees were assessed with 1,000 bootstrap replicates.

#### 2.2.4 Genome sequencing

A single colony of strain ZJU2 was inoculated into NB broth and incubated at 28 °C. When the culture reached an OD_600_ of 0.8, a 5 mL aliquot was centrifuged at 8,000 *× g* for 5 min to pellet the cells. The bacterial pellet was washed twice with sterile phosphate-buffered saline (PBS) and collected by centrifugation. The final pellet was sent to Novogene bioinformatics technology Co., Ltd. for whole-genome sequencing. The company performed sequencing using a combined strategy with the Illumina NovaSeq platform and the Oxford Nanopore PromethION platform. After quality control of the raw data, hybrid assembly and polishing were conducted using software such as Unicycler, ultimately yielding the complete circular genome sequence of strain ZJU2.

#### 2.2.5 Average nucleotide identity

Average nucleotide identity (ANI) is a crucial criterion for bacterial species delineation, where strains with an ANI value of >95% are classified as conspecific (Goris et al., 2007). The complete genome sequences of strain ZJU2 and the reference strain *P. ananatis* LMG 2665^T^ were submitted to the EzGenome online analysis platform^1^ for ANI calculation.

#### 2.2.6 Construction of core-genome phylogenomic tree

Representative strains of other species within the genus *Pantoea* were retrieved from the National Center for Biotechnology Information (NCBI) database. The core-genome phylogenomic tree was constructed using EasyCGTree V4.2 (Zhang et al., 2023). Specifically, HMMER was employed for homologous gene retrieval, MUSCLE5 for sequence alignment, trimAl for conservative region screening, and FastTree/IQ-TREE for phylogenetic tree construction.

### 2.3 Property determination of *Pantoea ananatis*

According to the method described by Tariq et al. (2023), we evaluated the bacterial swimming, phosphate solubilization, siderophore production, and indole-3-acetic acid (IAA) production of *P. ananatis* strains ZJU1-ZJU18.

#### 2.3.1 Swimming, methyl red, and voges proskauer tests

*Pantoea ananatis* strains ZJU1-ZJU18 were inoculated into MR-VP medium and cultured until the OD_600_ of the bacterial suspensions reached 0.8, corresponding to 2 × 10^8^ CFU/mL. Firstly, we spotted 3 μL of bacterial suspension onto NB medium containing 0.3% agar and incubated at 30 °C for 1 d to observe bacterial swimming. Then, we added VP detection solution A and solution B to the bacterial solution. If the color of the solution changed from pink to red within 2 min, the VP detection result was positive. In addition, we added the MR indicator solution to the bacterial solution. If the solution immediately turned red, it indicated that the MR detection result was positive.

#### 2.3.2 Enzyme activity determination

The 3% hydrogen peroxide solution was applied to the colonies of *P. ananatis* strains ZJU1-ZJU18. The appearance of bubbles indicated the presence of contact enzyme activity in the bacteria. The strains were cultured on amylase detection plates at 30 °C for 3 d, followed by the addition of a drop of Lugol’s iodine solution to the edge of the colonies. If no color change was observed, this suggested that the bacteria possess starch hydrolysis activity. *P. ananatis* strains ZJU1-ZJU18 were subsequently cultured on cellulase detection plates at 30 °C for 3 d. The formation of a transparent hydrolysis zones around the bacterial cells indicated cellulase production. Similarly, the strains were cultured on protease detection plates at 30 °C for 3 d, and the appearance of a transparent hydrolysis zones around the bacterial cells indicated protease production.

#### 2.3.3 Phosphate solubilization and siderophore activity detection

*P. ananatis* strains ZJU1-ZJU18 were cultured on Mongina inorganic phosphate and Mongina organic phosphate solid media at 30 °C for 3 d to evaluate their ability to dissolve phosphate. The phosphate solubilization zones around the colonies was observed, and the diameters of these zones were measured. Furthermore, *P. ananatis* strains ZJU1-ZJU18 were cultured on CAS agar plates at 30 °C for 3 d to assess siderophore production. The presence of yellow halos surrounding the colonies was recorded, and their diameters were measured accordingly.

#### 2.3.4 IAA synthesis detection

First, the IAA standard solutions with concentrations ranging from 0 to 100 μg/mL were mixed with equal volumes of Salkowski reagent and incubated in the dark for 30 min. The absorbance was measured at 530 nm, and a standard curve was generated using Microsoft Excel. Subsequently, strains ZJU1-ZJU18 were inoculated into LB broth supplemented with L-tryptophan (200 μg/mL) and incubated for 3 d. The cultures were centrifuged at 12,000 × *g* for 3 min to obtain the supernatant. A volume of 1,500 μL of the supernatant was mixed with an equal volume of Salkowski reagent. After standing in the dark for 30 min, the absorbance of the mixture was measured at 530 nm. Finally, the IAA concentration produced by each strain was calculated based on the standard curve.

### 2.4 Pathogenicity assay of *Pantoea ananatis*

#### 2.4.1 Leaf inoculation assay

Rice seeds were sterilized, germinated, and then three germinated seeds were transplanted into each pot (13 cm in diameter) filled with sterilized soil. The plants were cultivated in a greenhouse maintained at 28 °C with 70% relative humidity and a 16 h photoperiod. After 4 weeks of growth, the plants were used for inoculation. The bacterial suspensions (OD_600_ = 0.8) of *P. ananatis* strains ZJU1-ZJU18 were inoculated into rice leaves using the clip inoculation method (Luna et al., 2023). Sterile water was used as a negative control, and the bacterial suspension (OD_600_ = 0.8) of *Xoo* strain C2 was served as a positive control. Leaf disease symptoms were evaluated at 14 days post-inoculation (dpi). The experiments were performed with 3 replicates.

#### 2.4.2 Seed inoculation assay

The sterilized rice seeds were fully immersed in a suspension of *P. ananatis* (OD_600_ = 0.8) prepared in PBS and soaked at 28 °C for 2 h, with PBS used as a negative control. After soaking, the seeds were removed and gently blotted on sterile filter paper to remove excess liquid. They were then evenly placed on moist, sterile filter paper in Petri dishes, with 20 seeds per dish. The dishes were transferred to a growth chamber set at 28 °C under a 12 h light/12 h dark cycle for germination and growth. Germination symptoms were assessed after 3 d of cultivation.

### 2.5 Antibacterial activity of *Pantoea ananatis* against *Xoo in vivo*

Rice plants were cultivated as described in Section 2.4.1. After 4 weeks of growth, the plants were subjected to inoculation treatment. A mixture of sterile water and *Xoo* suspension served as the positive control, while sterile water alone was used as the negative control. In each independent experiment, at least ten rice leaves were treated for each treatment group. Lesion lengths on the leaves were measured at 14 dpi, and the experiments were independently repeated 3 times. In each independent experiment, at least ten rice leaves were treated for each treatment group. The disease control efficacy was calculated using the formula: Disease control efficacy = [(Lesion length of positive control – Lesion length of treatment) / Lesion length of positive control] × 100%.

#### 2.5.1 Antibacterial activity of multiple *Pantoea ananatis* strains

Resuspend the cell pellet of *P. ananatis* strains ZJU1-ZJU18 and *Xoo* strain C2 in sterile water and adjust the concentration to an OD_600_ value of 0.8. Equal volumes of the resuspended *P. ananatis* and *Xoo* suspensions were mixed. The mixed suspensions, positive control, and negative control were inoculated onto the leaves of Nipponbare rice using the leaf-clipping method.

#### 2.5.2 Effect of inoculation ratio on antibacterial activity

Resuspend the cell pellet of *P. ananatis* strains ZJU2 and *Xoo* strain C2 in sterile water and adjust the concentration to an OD_600_ value of 0.8. *Xoo* suspensions and *P. ananatis* suspensions were mixed at ratios of 1: 1, 10: 1, and 100: 1. The mixed suspensions, positive control, and negative control were inoculated onto the leaves of Nipponbare rice using the leaf-clipping method.

#### 2.5.3 Activity of strain ZJU2 against different *Xoo* strains

Resuspend the cell pellet of *P. ananatis* strains ZJU2 and *Xoo* strain C2, PXO99^A^ and N1 in sterile water and adjust the concentration to an OD_600_ value of 0.8. Equal volumes of the resuspended *P. ananatis* and *Xoo* suspensions were mixed. The mixed suspensions, positive control, and negative control were inoculated onto the leaves of Nipponbare rice using the leaf-clipping method.

#### 2.5.4 Activity comparison between rice varieties

Resuspend the cell pellet of *P. ananatis* strains ZJU2 and *Xoo* strain C2 in sterile water and adjust the concentration to an OD_600_ value of 0.8. Equal volumes of the resuspended *P. ananatis* and *Xoo* suspensions were mixed. The mixed suspensions, positive control, and negative control were separately inoculated onto the leaves of Nipponbare rice and 9,311 rice using the leaf-clipping method.

### 2.6 Antibacterial activity of *Pantoea ananatis* against Xoo *in vitro*

The antibacterial activity of *P. ananatis* was evaluated with minor modifications with minor modifications to the method described by Xie et al. (2023). First, 1 mL of *Xoo* strain C2 suspension (OD_600_ = 0.8) was mixed with 7 mL of semi-solid NA medium (0.8% agar) to prepare the double-layer *Xoo* plate. Subsequently, 3 *μ*L of *P. ananatis* suspension (OD_600_ = 0.8) was spotted onto the double-layer *Xoo* plate. After the suspension was absorbed, the plates were incubated at 30 °C for 48 h, and the formation of inhibition zones was observed and recorded.

To assess the antibacterial activity of *P. ananatis* cell-free fermentation broth against *Xoo*, strains ZJU1-ZJU18 were inoculated into 100 mL of NB broth and incubated with shaking at 30 °C for 72 h. The culture was centrifuged at 12,000 × *g* for 10 min, and the supernatant was filtered through a 0.22 *μ*m sterile membrane to obtain the cell-free fermentation broth. A hole (8 mm in diameter) was punched in the center of the *Xoo* double-layer plate, and 100 μL of the cell-free fermentation broth was added to each hole. The plates were incubated at 30 °C for 48 h, and the formation of inhibition zones was observed.

### 2.7 Bacterial growth curve assay

The concentrations of *Xoo* strain C2 and *P. ananatis* strain ZJU2 suspensions were adjusted to 1.0 × 10^8^ CFU/mL. Add 100 μL *Xoo* and *P. ananatis* to 5 mL NB broth, respectively. Subsequently, the culture was incubated at 30 °C for 48 h. The OD_600_ was measured at 3 h intervals, and the growth curves were generated accordingly. The specific growth rate (μ) was calculated based on the growth curve. Data points with optical density (OD_600_) values between 0.1 and 0.6 were selected, as within this range the OD value exhibits a linear relationship with cell concentration. The natural logarithm was taken of these OD values to obtain ln(OD). Using cultivation time as the independent variable and ln(OD) as the dependent variable, linear regression analysis was performed on the data points corresponding to the exponential growth phase. The resulting linear equation is y = *a*x + b, where the slope a represents the specific growth rate μ (h^−1^).

### 2.8 Flow cytometry analysis

To construct the fluorescent strain Pa (GFP), the plasmid pRADK-GFP was introduced into *P. ananatis* strain ZJU2. The concentrations of *Xoo* strain C2 and *P. ananatis* strain Pa (GFP) suspensions were adjusted to 1.0 × 10^8^ CFU/mL. Subsequently, the two strains were co-inoculated into 5 mL of NB medium at initial volume ratios of 1: 1, 10: 1, and 100: 1. For the monoculture control, an equivalent volume of Pa (GFP) suspension was inoculated into 5 mL of NB broth, with sterile NB broth added in place of the *Xoo* suspension. After incubation at 30 °C with shaking for 72 h, the proportion of GFP-positive cells in each suspension was determined by flow cytometry. The relative abundance of *P. ananatis* in the co-culture system was calculated as follows: Relative abundance of *P. ananatis* = [(Proportion of GFP-positive cells in co-culture)/(Proportion of GFP-positive cells in monoculture)] × 100%.

### 2.9 Bacterial count of culture medium

To differentiate between *P. ananatis* and *Xoo* in co-culture broth, we introduced plasmid pBBR1MCS RFP carrying Km resistance gene into *Xoo* strain C2 to construct marker strain Xoo (RFP), and vector pBBR1MCS-5 carrying Gen resistance gene into *P. ananatis* strain ZJU2 to construct marker strain Pa (Gen). The population densities of *Xoo* and *P. ananatis* in co-culture broth could be independently quantified by counting colony-forming units on selective media amended with either Km or Gen.

The bacterial suspensions of Xoo (RFP) and Pa (Gen) were adjusted to a concentration of 1.0 × 10^8^ CFU/mL. Subsequently, Xoo (RFP) and Pa (Gen) were co-inoculated into NB broth, NL broth, and CL broth at initial volume ratios of 1: 1, 10: 1, and 100: 1. The cultures were incubated at 30 °C for 3 d, after which the mixed bacterial suspensions were subjected to 10-fold serial dilutions. Aliquots of 2.5 μL from each dilution were spotted onto NB, NL, and CL agar plates supplemented with either Km or Gen. The plates were incubated at 30 °C for 3 d, and the number of colonies was counted.

### 2.10 Spot assay

The bacterial suspensions of Xoo (RFP) and Pa (Lux) were adjusted to a concentration of 1.0 × 10^8^ CFU/mL. On the bottom of a NA plate containing Km, a inverted “V” shape was drawn with a marker to guide inoculation. Then, 2.5 μL of Pa (Lux) and Xoo (RFP) suspensions were inoculated separately along the guide lines on the NA plate. The plate was incubated upright at 28 °C for 48 h, after which the growth of *P. ananatis* and *Xoo* colonies was observed.

### 2.11 Bacterial count of rice leaves

The cultivation of Nipponbare rice plants followed the method described in Section 2.4.1. Prior to inoculation, the bacterial suspensions of Xoo (RFP) and Pa (Gen) were adjusted to a concentration of 1.0 × 10^8^ CFU/mL. Rice leaves were inoculated with Xoo (RFP) alone, Pa (Gen) alone, or a mixture of both using the leaf-clipping method. Samples were subsequently collected at 1, 3, 7, and 14 dpi. After the surface sterilization of rice leaves, the absence of bacterial growth in the final rinse water confirmed the effectiveness of the sterilization process.

For qPCR analysis, six leaves were collected per treatment at each time point. Following surface sterilization, total genomic DNA was extracted from the tissues using the QIAGEN PowerSoil DNA Extraction Kit. The bacterial populations were quantified by qPCR with species-specific primers, and absolute counts were calculated based on a standard curve.

For the drop plate assay, three leaves were collected per treatment at each time point. After the same surface sterilization procedure, leaves were homogenized. The resulting leaf homogenates were serially diluted tenfold, and 2 μL aliquots of each dilution were spotted onto NA plates supplemented with the appropriate antibiotics. After incubation at 30 °C for 72 h, the number of colony-forming units was counted to determine the viable bacterial population.

### 2.12 Leaf bioluminescence imaging assay

The leaf bioluminescence imaging assay was conducted with slight modifications to the previously described method by Xu et al. (2022). The pBBR1MCS2-*luxCDABE* plasmid was successfully introduced into *P. ananatis* strain ZJU2 to generate the luminescent strain Pa (Lux). Subsequently, *Xoo* strain C2 and *P. ananatis* strain Pa (Lux) were adjusted to a concentration of 1.0 × 10^8^ CFU/mL.

Nipponbare rice leaves were inoculated with a mixed suspension of *P. ananatis* and *Xoo* using the leaf-clipping method. For the control treatment, leaves were inoculated with a suspension of *P. ananatis* mixed with an equivalent volume of NB broth. At 1, 3, 7, and 14 dpi, the rice leaves were collected. After the surface sterilization of rice leaves, the absence of bacterial growth in the final rinse water confirmed the effectiveness of the sterilization process. The bioluminescent signals were monitored using a single-photon imaging system equipped with a Photek HRPCS5 camera (Photek Ltd., East Sussex, United Kingdom) with an acquisition time of 5 min. Finally, the quantitative data were exported using Image32 software (version 3.6.2) for analysis.

### 2.13 16S amplicon sequencing of the phyllosphere endophytic microbiome

Nipponbare rice leaves were collected at 1 and 14 dpi under four treatment conditions: uninoculated control, inoculated with *Xoo*, inoculated with *P. ananatis*, and co-inoculated with both *Xoo* and *P. ananatis*. Leaf samples were flash-frozen in liquid nitrogen, and 0.2 g of each sample was ground into a fine powder using a leaf tissue grinder. Samples from different treatment groups should be randomly mixed as much as possible for library construction and sequencing, so as to reduce potential batch effects. Total genomic DNA was extracted using the QIAGEN PowerSoil DNA Isolation Kit. Subsequently, the V4-V5 hypervariable region of the bacterial *16S rRNA* gene was amplified using region-specific primers. The resulting amplicons were purified, quantified, and subjected to paired-end sequencing by Parsono Biotechnology Co., Ltd. (Shanghai, China). Raw sequence data were processed using the DADA2 pipeline, which included primer trimming, quality filtering, denoising, merging of paired reads, and removal of chimeric sequences. After quality control, unique sequences were identified as amplicon sequence variants (ASVs). A feature table was generated based on the abundance of ASVs across samples. For alpha/beta diversity analysis, all samples were uniformly rarefied to 95% of the sequence number of the sample with the lowest sequencing depth. Downstream data analyses were conducted using the GenesCloud online platform.^2^

## 3 Results

### 3.1 Identification of *Pantoea ananatis*

**Figure 1:**
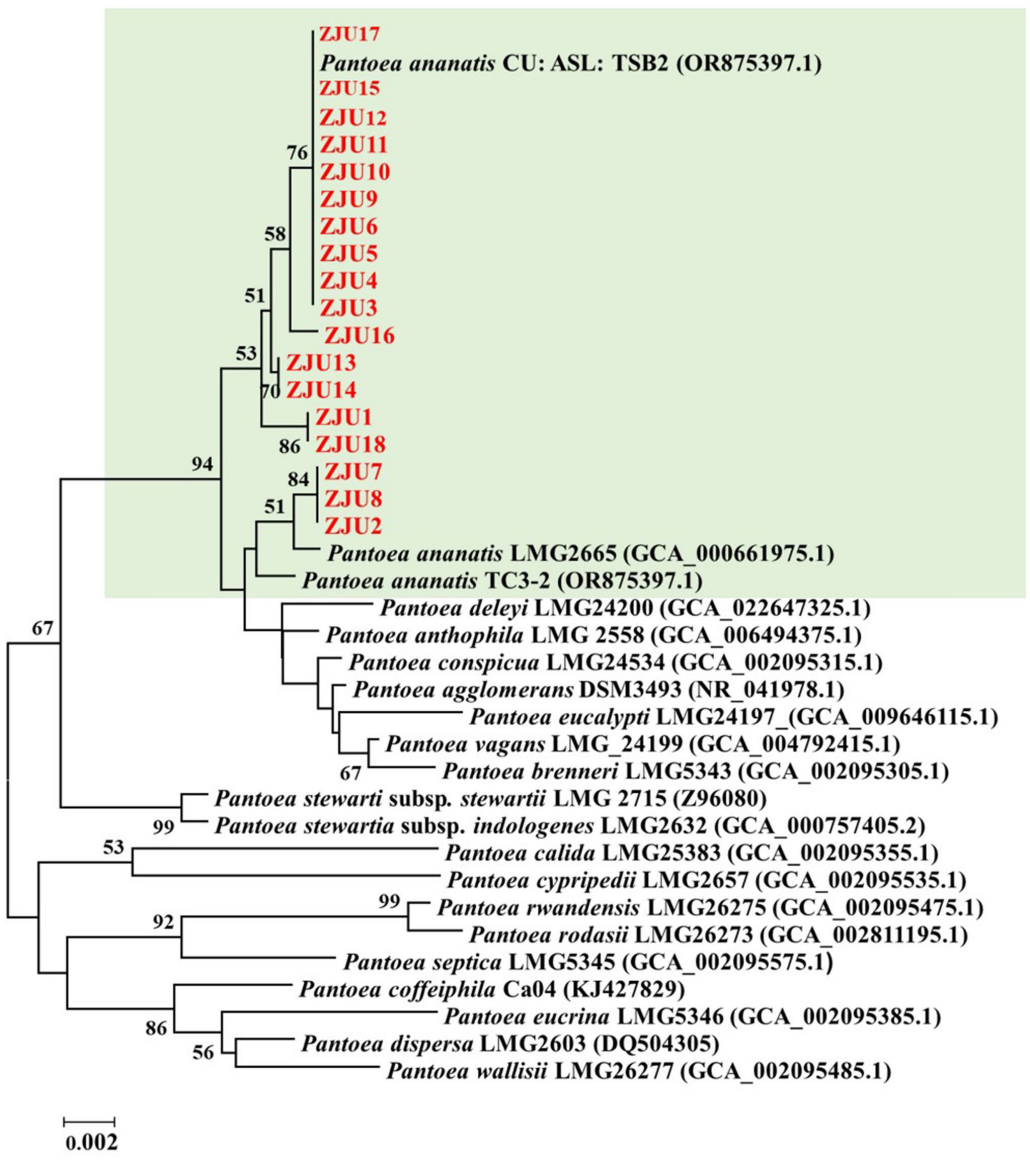
Phylogenetic tree based on *16S rRNA* gene sequence analysis. Numbers at the nodes represent bootstrap support values estimated from 1,000 replicates. Only values above 50% are displayed.

Multiple *Pantoea* isolates were obtained from each sampling site. The *16S rRNA* gene sequences of all isolates were aligned and compared, and isolates exhibiting identical or nearly identical sequences (>99.5% similarity) were grouped into the same operational taxonomic unit (OTU). From each OTU at each sampling site, one isolate was selected as a representative strain for subsequent molecular phylogenetic and functional analyses. These isolates were designated ZJU1 through ZJU18, and their corresponding sequencing data were deposited into the NCBI database (Supplementary Table S3). The phylogenetic tree based on *16S rRNA* gene sequence analysis revealed that the isolated *Pantoea* strains belonged to *P. ananatis* (Figure 1).

**Figure 2:**
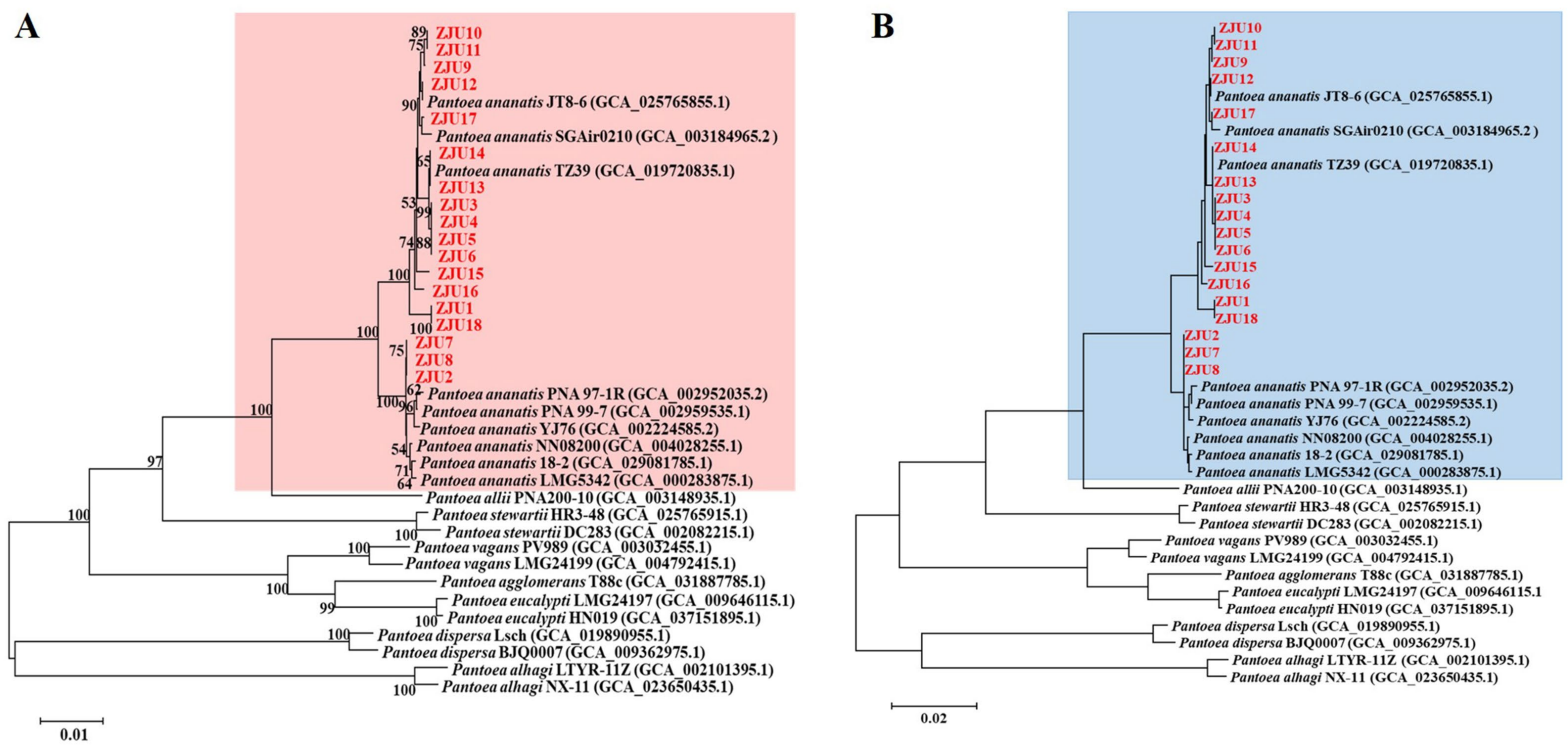
MLSA phylogenetic trees constructed based on the conserved genes *atpD*, *gyrB*, *fusA*, and *rpoB*. **(A)** Neighbor-joining method. **(B)** Maximum likelihood method. Numbers at the nodes represent bootstrap support values estimated from 1,000 replicates. Only values above 50% are displayed.

MLSA can infer phylogenetic relationships by integrating sequence information from multiple conserved genes, thereby improving resolution and accuracy (Brady et al., 2008; Tambong, 2019; Suleimanova et al., 2021; Guedj-Dana et al., 2022). Following the recommended criteria for species demarcation outlined in the study by De Armas et al. (2022), we found that the core clustering patterns of the two MLSA phylogenetic trees constructed using the NJ and ML methods were highly congruent. Strains ZJU1-ZJU18 formed a major clade with members of the *P. ananatis* group (Figure 2). The relationships among other species (*P. allii*, *P. stewartii*, *P. vagans*, *P. agglomerans*, etc.) were also largely consistent between the two methods. In the MLSA phylogeny based on the NJ method, the major clade comprising strains ZJU1-ZJU18 and the *P. ananatis* group received a bootstrap support value of 100. Within this clade, ZJU17 clustered with *P. ananatis* strain JT8-6 with a support value of 90, while ZJU14 clustered with *P. ananatis* strain TZ39 with a support value of 65 (Figure 2A). Therefore, we concluded that strains ZJU1-ZJU18 are all *P. ananatis.*

Whole-genome sequencing of the representative strain ZJU2 revealed a genome comprising one chromosome and one plasmid. The chromosome is 4,472,381 bp in length with a G + C content of 53.59%, while the plasmid is 365,824 bp with a G + C content of 52.37%. The complete genome sequence has been deposited in the NCBI database under accession number SUB15922611. A circular map of the chromosome is presented in Supplementary Figure S1, displaying from outermost to innermost: genomic coordinates, protein-coding genes, functional annotations, ncRNAs, genomic GC content, and GC skew.

To taxonomically classify ZJU2, we performed ANI analysis against *P. ananatis* strain LMG 2665^T^. The resulting ANI value was 99.28%, substantially above the 95.00% threshold widely used for species delineation (Goris et al., 2007). Therefore, based on genomic evidence, strain ZJU2 was identified as *P. ananatis*.

**Figure 3:**
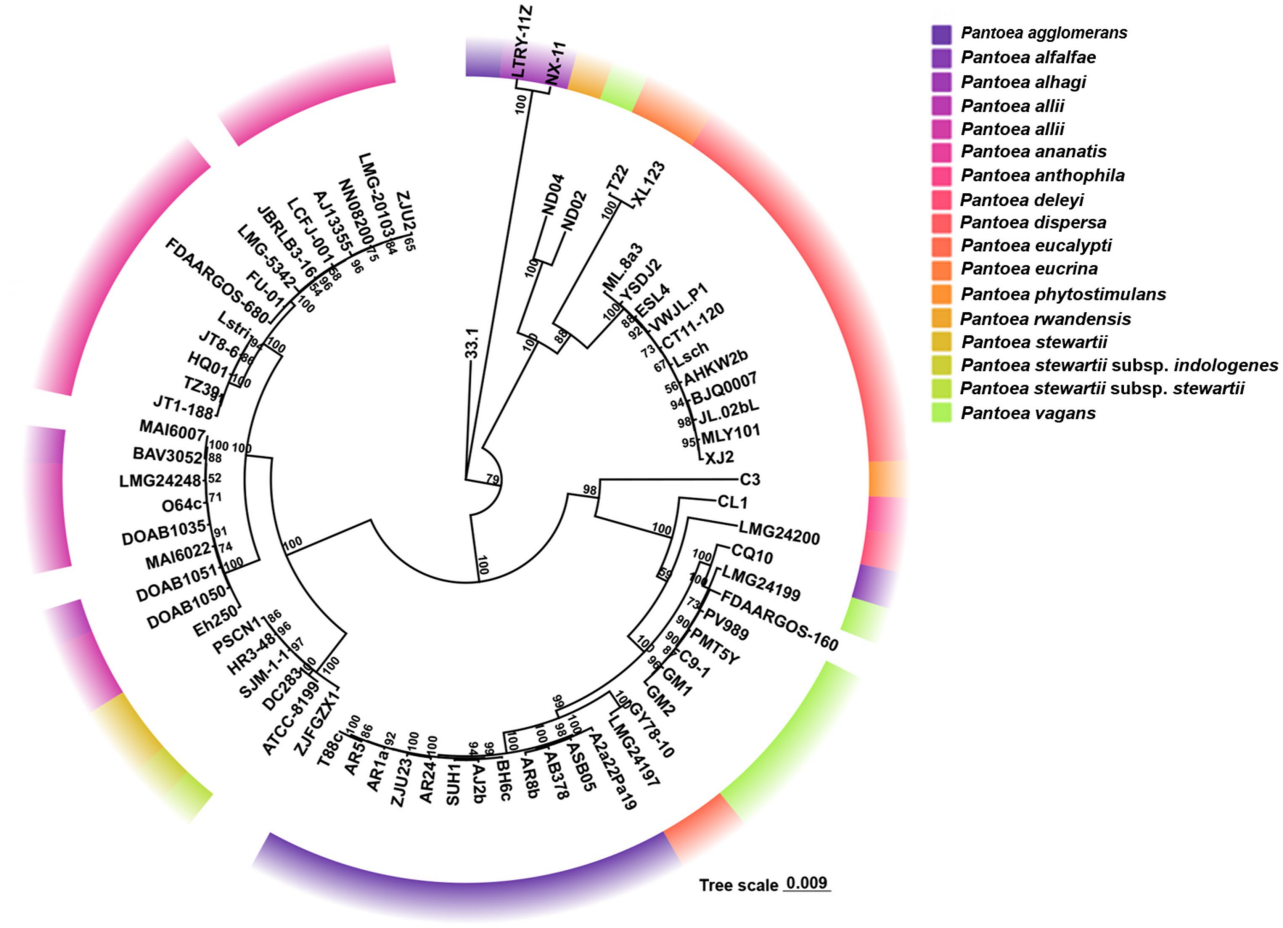
Core-genome phylogenomic tree of the genus *Pantoea.* Distinctly colored clades correspond to discrete species of the genus *Pantoea*, as delineated in the legend. The tree scale (0.009) denotes the genetic distance. Values adjacent to nodes represent bootstrap support (derived from 1,000 replicates), and only those exceeding 50 are depicted in the figure.

Based on core-genome phylogenomic analysis, we clarified the taxonomic status of strain ZJU2 and its evolutionary relationships within the species *P. ananatis* (Figure 3). The tree revealed a well-resolved phylogeny with strong support at key nodes. Critically, all *P. ananatis* strains, including ZJU2, formed a monophyletic clade with maximum (100%) bootstrap support, providing robust genomic evidence that ZJU2 belongs to this species. Within the *P. ananatis* clade, strain ZJU2 clustered most closely with the reference strain LMG 20103. At the genus level, *P. ananatis* was clearly distinguished from other species such as *P. stewartii* and *P. dispersa*, each forming independent, well-supported evolutionary branches, further validating the reliability of the core-genome phylogenomic analysis performed here.

### 3.2 Characterization of *Pantoea ananatis* traits

**Figure 4:**
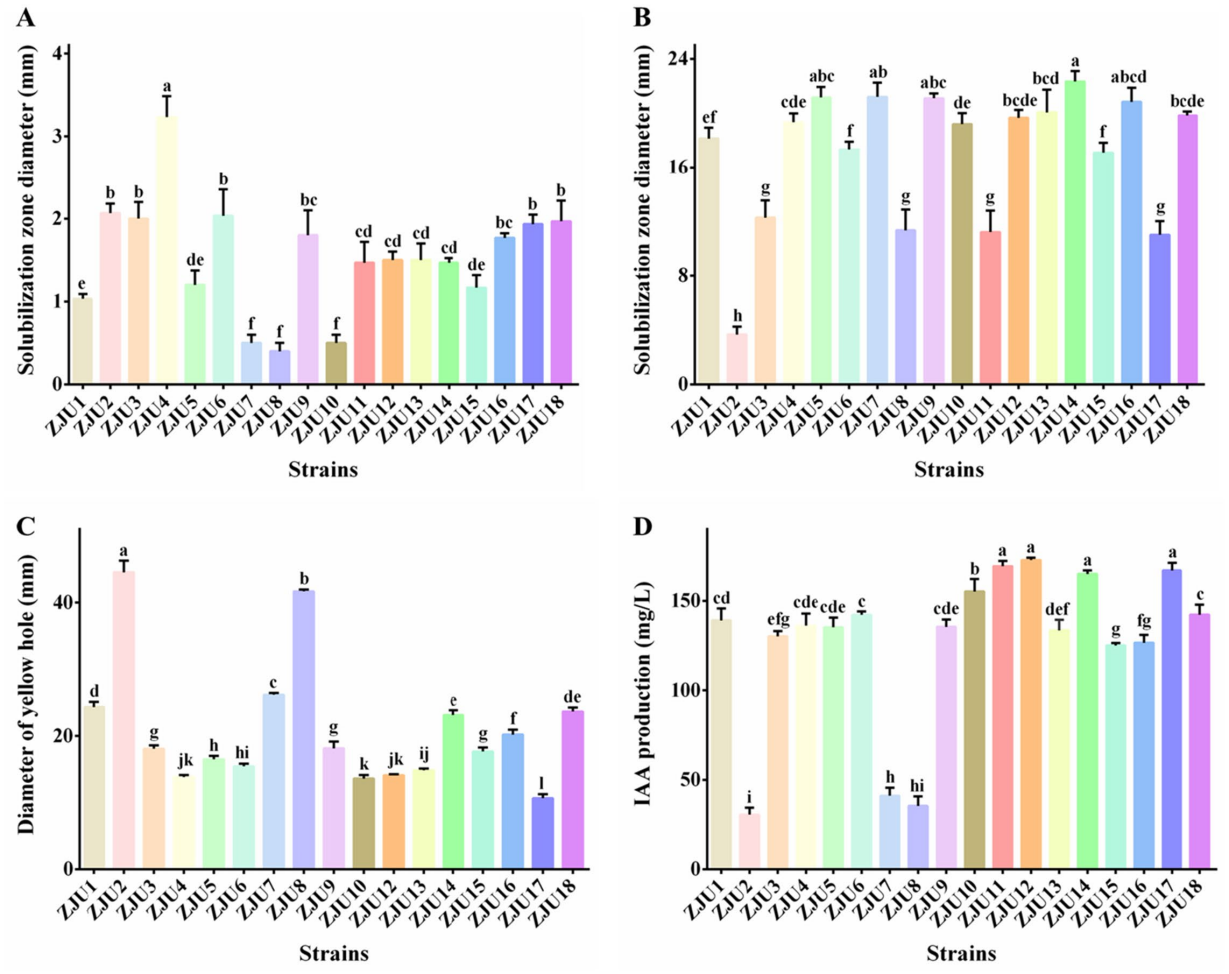
Determination of the promoting properties of ZJU1–ZJU18. **(A)** Inorganic phosphorus solubilization. **(B)** Organic phosphorus solubilization. **(C)** Siderophore production. **(D)** IAA production. Statistical differences were analyzed by one-way ANOVA. Different lowercase letters within the same time point and assay indicate significant differences between treatments (*p* < 0.05).

This study found that the VP test produced positive results for *P. ananatis* strains ZJU1-ZJU18, while the MR test was negative. Enzyme activity assays revealed that strains ZJU1-ZJU18 did not produce catalase, cellulase, protease, or amylase. Furthermore, all strains ZJU1-ZJU18 exhibited good swimming (Supplementary Figure S2A). Besides, strain ZJU18 displayed the strongest swimming ability, with a swimming diameter of 63.67 mm, whereas strain ZJU10 showed the weakest swimming ability, with a swimming diameter of 15.33 mm (Supplementary Table S4). Bacteria possessing plant growth-promoting (PGP) traits can promote plant growth. Our research found that *P. ananatis* strains ZJU1-ZJU18 could form clear halos on both inorganic and organic phosphate plates, demonstrating their ability to dissolve both inorganic and organic phosphates (Supplementary Figures S2B,C). Quantitative analysis indicated that strain ZJU4 exhibited the highest inorganic phosphate solubilization capacity, with a halo diameter of 3.23 mm, while strain ZJU14 showed the strongest organic phosphate solubilization, with a halo diameter of 22.33 mm (Figures 4A,B). Except for strain ZJU11, other isolated *P. ananatis* strains formed characteristic yellow halos on CAS plates (Supplementary Figure S2D), indicating siderophore production. Strain ZJU2 displayed the strongest siderophore synthesis capacity, with a halo diameter of 44.53 mm (Figure 4C). Besides, the standard curve for IAA quantification was established using spectrophotometry, yielding the equation: y = 0.01x + 0.1089 (*R*^2^ = 0.9794). Our study demonstrated that all *P. ananatis* strains were capable of synthesizing IAA. Notably, strains ZJU12, ZJU11, ZJU17, and ZJU14 exhibited significantly higher IAA production levels, with yields of 172.78 μg/mL, 169.30 μg/mL, 166.98 μg/mL, and 164.97 μg/mL, respectively (Figure 4D).

### 3.3 *Pantoea ananatis* can suppress rice bacterial leaf blight in greenhouse

**Figure 5:**
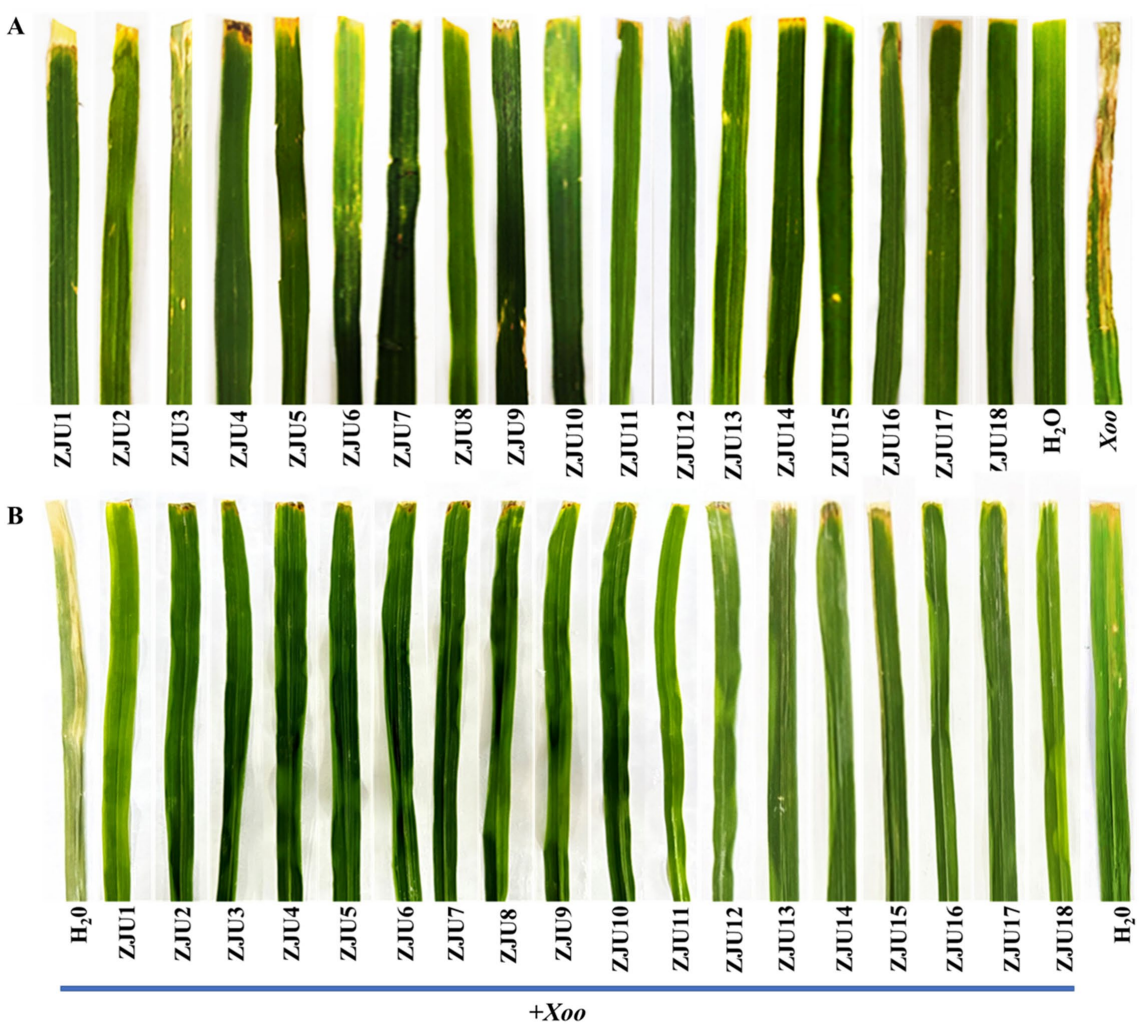
Rice leaf symptoms observed at 14 dpi with different bacteria. **(A)** Inoculation with *P. ananatis* strains ZJU1–ZJU18. **(B)** Co-inoculation with *P. ananatis* strains ZJU1–ZJU18 and *Xoo* strain C2.

Leaf-clipping assays showed that rice leaves inoculated with sterile water exhibited no significant lesions. In contrast, leaves inoculated with *Xoo* strain C2 developed characteristic yellowish-brown lesions that expanded along the leaf veins. However, Rice leaves inoculated with *P. ananatis* strains ZJU1-ZJU18 exhibited no significant disease symptoms, with only minor chlorotic spots observed at the inoculation sites. Based on these results, we concluded that *P. ananatis* strains ZJU1-ZJU18 did not induce leaf blight symptoms under the tested conditions (Figure 5A). Furthermore, we observed that these strains did not inhibit the germination of rice seeds (Supplementary Figure S3; Supplementary Table S5).

**Figure 6:**
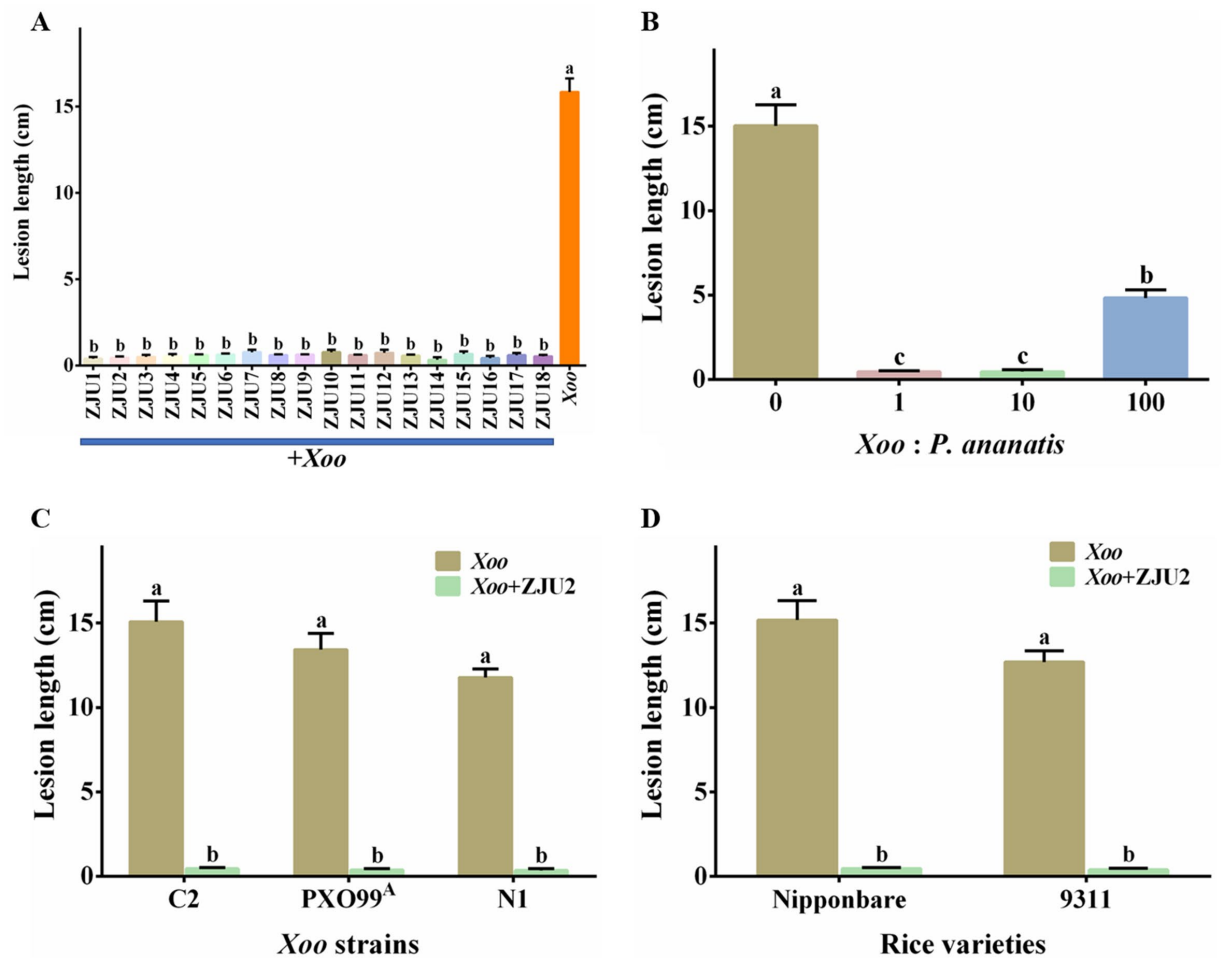
Antibacterial activity of *P. ananatis* against *Xoo in vivo*. **(A)** Antibacterial activity of multiple *P. ananatis* strains. **(B)** Effect of inoculation ratio on antibacterial activity. **(C)** Activity of strain ZJU2 against different *Xoo* strains. **(D)** Activity comparison between rice varieties. Statistical differences were analyzed by one-way ANOVA. Different lowercase letters within the same time point and assay indicate significant differences between treatments (*p* < 0.05).

To investigate whether different *P. ananatis* strains consistently exhibit inhibitory effects on the development of rice bacterial leaf blight, we co-inoculated *P. ananatis* strains ZJU1-ZJU18 individually with *Xoo* strain C2 onto the leaves of japonica rice cultivar Nipponbare. The results revealed that rice leaves inoculated with *Xoo* and sterile water developed symptoms of yellowing and desiccation. Conversely, only slight chlorosis spots were observed on the rice leaves co-inoculated with *P. ananatis* and *Xoo* (Figure 5B). The lesion length on leaves inoculated with *Xoo* and sterile water was 15.84 cm, whereas the lesion lengths on leaves co-inoculated with *P. ananatis* and *Xoo* ranged from 0.33 cm to 0.77 cm (Figure 6A). *P. ananatis* strains ZJU1-ZJU18 significantly reduced the lesion length caused by *Xoo* strain C2, with a disease inhibition rate ranging from 95.14 to 97.92%.

To determine whether the inhibitory effect of *P. ananatis* on rice bacterial leaf blight is dependent on the *Xoo* strain, leaves of the japonica rice cultivar Nipponbare were co-inoculated with *P. ananatis* strain ZJU2 and different *Xoo* strains at a 1: 1 ratio. When inoculated alone, *Xoo* strains C2, PXO99^A^, and N1 produced lesion lengths of 15.08 cm, 13.43 cm, and 11.78 cm, respectively (Figure 6B). In contrast, co-inoculation with *P. ananatis* ZJU2 markedly reduced lesion development to 0.33 cm, 0.34 cm, and 0.37 cm, corresponding to disease suppression rates of 97.81, 97.47, and 96.86%, respectively. The results indicated that *P. ananatis* strain ZJU2 could effectively inhibit rice bacterial leaf blight caused by different *Xoo* strains in Nipponbare rice. Additionally, to determine whether the inhibitory effect of *P. ananatis* on rice bacterial leaf blight is influenced by the inoculum ratio of *Xoo* to *P. ananatis*, leaves of the japonica rice cultivar Nipponbare were inoculated with *Xoo* strain C2 and *P. ananatis* strain ZJU2 at different ratios. When inoculated alone, *Xoo* strain C2 caused lesions with a mean length of 15.03 cm. In contrast, co-inoculation at *Xoo: P. ananatis* ratios of 1: 1, 10: 1, and 100: 1 resulted in lesion lengths of 0.45 cm, 0.46 cm, and 4.83 cm, respectively (Figure 6C). The corresponding disease suppression rates were 97.00, 96.94, and 67.87%. The results showed that the lesion inhibition rates of *P. ananatis* were comparable when the inoculation ratios of *Xoo* to *P. ananatis* were 1: 1 and 10: 1, whereas the inhibition rate decreased significantly when the ratio increased sharply to 100: 1.

Furthermore, to determine whether the inhibitory effect of *P. ananatis* on rice bacterial leaf blight is cultivar-dependent, we performed co-inoculation experiments with the *Xoo* strain C2 and *P. ananatis* strain ZJU2. The results showed that in the japonica rice cultivar Nipponbare, the lesion length caused by *Xoo* alone was 15.18 cm (Figure 6D). In contrast, co-inoculation with *Xoo* and *P. ananatis* resulted in a lesion length of only 0.45 cm, corresponding to a disease inhibition rate of 93.23%. Similarly, in the indica rice cultivar 9,311, the lesion length caused by *Xoo* alone was 12.69 cm, while co-inoculation with *Xoo* and *P. ananatis* reduced the lesion length to 0.39 cm, also yielding a disease inhibition rate of 96.93%. The results confirmed that *P. ananatis* strain ZJU2 exhibited a significant inhibitory effect against *Xoo* strain C2 across different rice varieties, with the disease inhibition rate exceeding 90.00% in all cases, which indicated that it possesses a broad spectrum antibacterial activity. In conclusion, these results indicated that *P. ananatis* has strong biocontrol potential and can effectively mitigate the damage inflicted by *Xoo* on rice.

### 3.4 *Pantoea ananatis* inhibits *Xoo* through nutritional competition in broth

Double-layer plate experiments indicated that while *P. ananatis* cells produced a faint inhibition zone against *Xoo*, its cell-free fermentation supernatant failed to generate any zone. This finding prompted further investigation into the underlying ecological interaction mechanisms.

**Figure 7:**
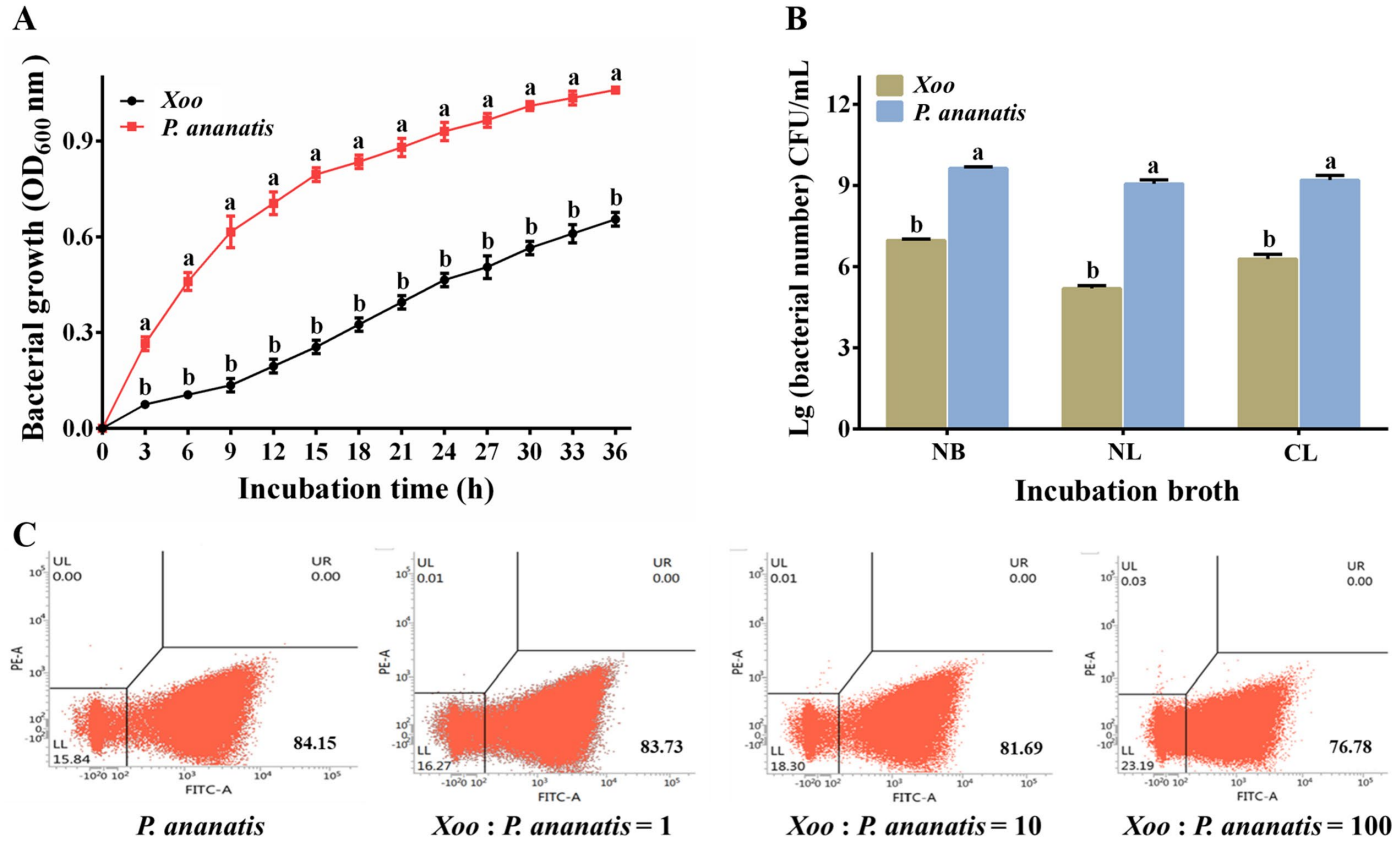
Growth advantages and nutritional competition of *P. ananatis* and *Xoo* in culture medium. **(A)** Growth curves of *Xoo* and *P. ananatis*. **(B)** The populations of *Xoo* and *P. ananatis* of co-culture in NB, NL, and CL broth. **(C)** Flow cytometry analysis of *Xoo* and *P. ananatis* co-cultured at different initial ratios (1: 1, 10: 1, and 100: 1). Statistical differences were analyzed by one-way ANOVA. Different lowercase letters within the same time point and assay indicate significant differences between treatments (*p* < 0.05).

Our study first investigated whether *P. ananatis* inhibits *Xoo* via nutrient competition. Growth curve analysis demonstrated that both *Xoo* and *P. ananatis* exhibited increased growth over time. At each corresponding time point, the OD_600_ value of *P. ananatis* was consistently higher than that of *Xoo* (Figure 7A). Through calculation, we determined that the fitted growth rate of *P. ananatis* was 0.140 h^−1^, while that of *Xoo* was 0.063 h^−1^. This demonstrated that the growth rate of *P. ananatis* is significantly faster than that of *Xoo*.

The growth of Pa (Gen) showed no significant difference compared to the wild-type strain ZJU2, and the growth of Xoo (RFP) was comparable to wild-type strain C2 (Supplementary Figure S4). Co-cultures of Pa (Gen) and Xoo (RFP) were established in different broth, and plate counting was employed to enumerate *Xoo* and *P. ananatis* populations (Table 1; Figure 7B). Through calculation, we found that in NB broth, *P. ananatis* accounted for 99.81% of the total population, while the *Xoo* population constituted 0.19%. In NL broth, *P. ananatis* represented 99.99%, with *Xoo* only making up 0.01%. In CL broth, *P. ananatis* comprised 98.82%, and *Xoo* accounted for 1.18%.

Pa(GFP), which showed no significant growth differences compared to the wild-type strain ZJU2, was used as a representative strain in flow cytometry experiments to determine the proportion of *P. ananatis* in mixed cultures with varying initial ratios of *Xoo* to *P. ananatis* (Supplementary Figure S4B). When *Xoo* and *P. ananatis* were co-cultured at initial ratios of 1: 1, 10: 1, and 100: 1, the proportions of fluorescent bacteria in the mixed cultures were 83.73, 81.69, and 76.78%, respectively (Figure 7C). Based on these data, the calculated proportions of *P. ananatis* in the mixed cultures were 99.50, 96.78, and 90.53% for the 1: 1, 10: 1, and 100: 1 initial ratios, respectively. These results consistently supported that *P. ananatis* inhibits *Xoo* through nutritional competition in broth. In addition, the spot assay provided visual evidence of a competitive interaction. We observed that the size of the *Xoo* colony decreased as the distance to the *P. ananatis* spot diminished (Supplementary Figure S5). This spatial pattern of growth inhibition is consistent with a scenario where *P. ananatis*, potentially due to its faster growth rate, outcompetes *Xoo* for limited nutrients in this assay system. It should be noted that the nutrient environment within plant leaves may differ from this *in vitro* condition.

### 3.5 *Pantoea ananatis* inhibits the colonization of *Xoo* on rice leaves

**Figure 8:**
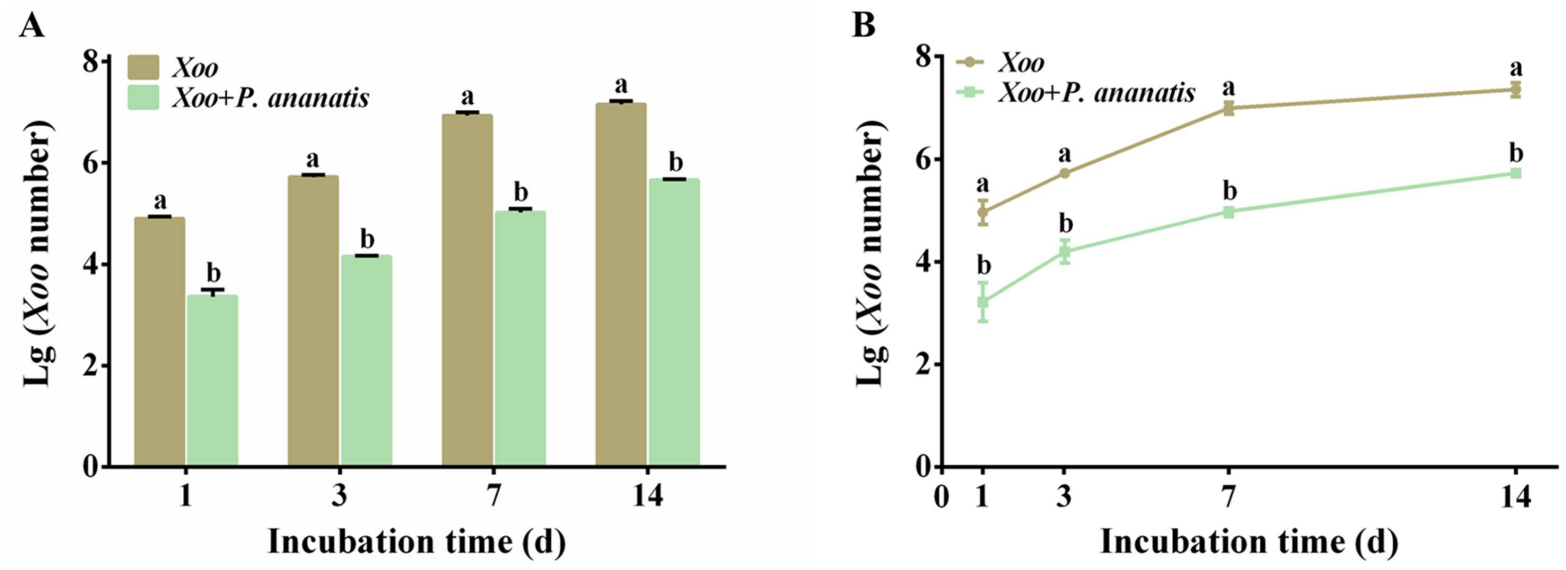
Quantification of *Xoo* in rice leaves at 1, 3, 7, and 14 dpi with *Xoo* alone or co-inoculation with *P. ananatis*. **(A)** Quantify *Xoo* through qPCR analysis. **(B)** Quantify *Xoo* through plate counting analysis. Statistical differences were analyzed by one-way ANOVA. Different lowercase letters within the same time point and assay indicate significant differences between treatments (*p* < 0.05).

qPCR analysis showed that the standard curve for absolute quantification of *Xoo* was y = −4.0034x + 41.004 (*R*^2^ = 0.994). qPCR analysis and plate counting experiments confirmed that co-inoculation with *P. ananatis* significantly reduced the population of *Xoo* in rice leaves across all time points examined (Figures 8A,B; Table 2). Compared with leaves inoculated solely with *Xoo*, the infection rate of *Xoo* on leaves was reduced by 96.78 to 99.00% following co-inoculation with *Xoo* and *P. ananatis.* These results indicated that the quantity of *Xoo* in leaves inoculated solely with *Xoo* was significantly higher than in leaves co-inoculated with *Xoo* and *P. ananatis* at all time points. Thus, *P. ananatis* significantly inhibits *Xoo* colonization on rice leaves.

**Table 2:** Population dynamics of *Xoo* in rice leaves and the inhibitory effect of *P. ananatis* co-inoculation.

Inoculation time (d)	*Xoo* population in leaves (CFU/mL)			
	qPCR		Plate count	
	*Xoo+Pa*	*Xoo*	*Xoo+Pa*	*Xoo*
1	2.29 × 10^3^	7.76 × 10^4^	2.33 × 10^3^	1.00 × 10^5^
3	1.41 × 10^4^	5.25 × 10^5^	1.67 × 10^4^	5.33 × 10^5^
7	1.05 × 10^5^	8.51 × 10^6^	1.00 × 10^5^	1.00 × 10^7^
14	4.57 × 10^5^	1.42 × 10^7^	5.33 × 10^5^	2.33 × 10^7^

### 3.6 *Xoo* enhances the colonization of *Pantoea ananatis* in rice leaves

**Figure 9:**
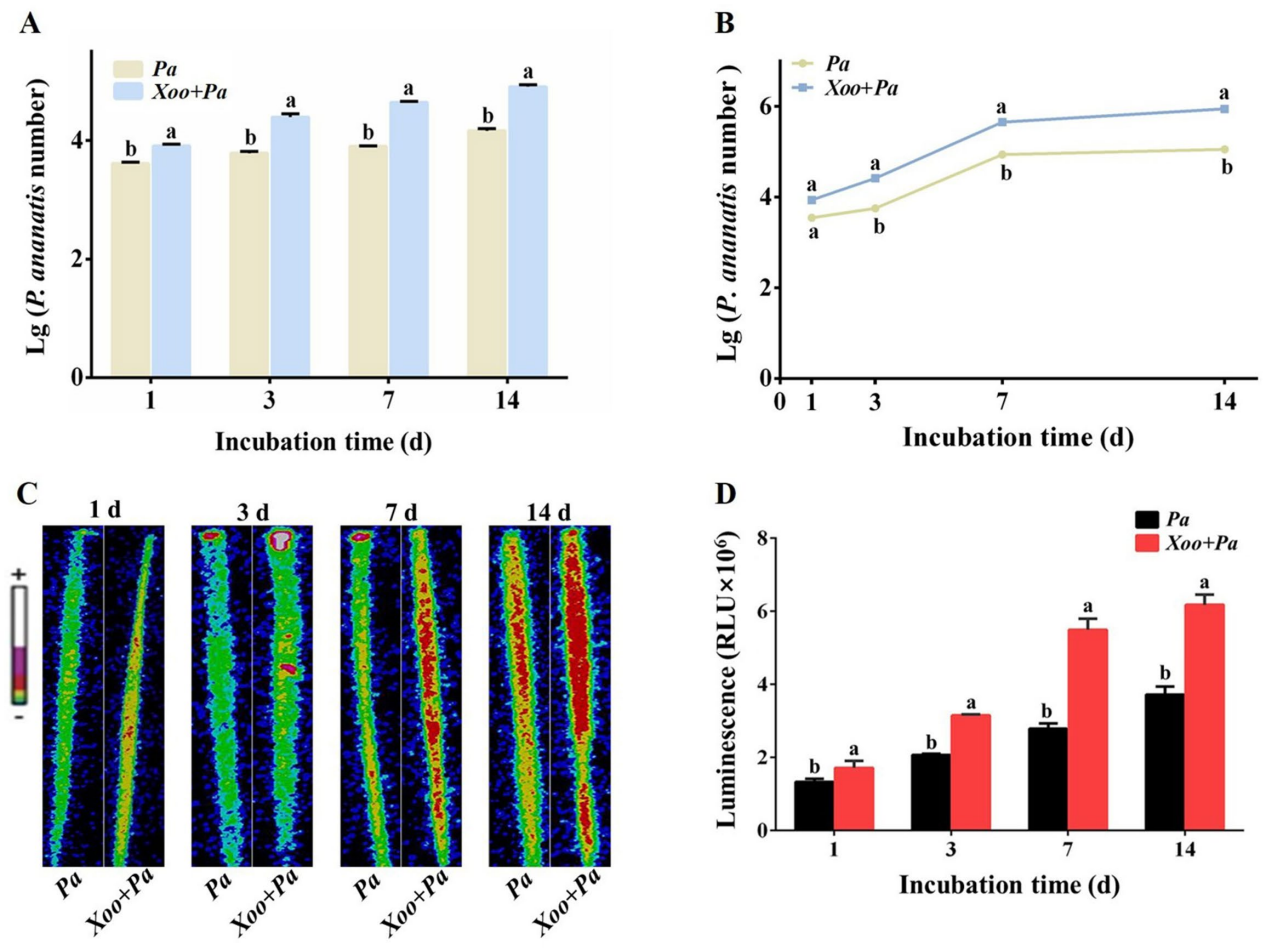
Quantification of *P. ananatis* in rice leaves at 1, 3, 7, and 14 dpi with *P. ananatis* alone or co-inoculation with Xoo. **(A)** Quantify *P. ananatis* through qPCR analysis. **(B)** Quantify *P. ananatis* through plate counting analysis. **(C)** Luminous signal image. **(D)** Luminous signal intensity. +: Strong; -: Weak. Scale bar = 1 cm. Statistical differences were analyzed by one-way ANOVA. Different lowercase letters within the same time point and assay indicate significant differences between treatments (*p* < 0.05).

Based on both qPCR (standard curve: y = −4.767x + 46.209, *R*^2^ = 0.989) and plate counting assays, the population of *P. ananatis* was consistently and significantly higher in leaves co-inoculated with *Xoo* compared to leaves inoculated with *P. ananatis* alone at all time points (Figures 9A,B; Table 3). Specifically, the presence of *Xoo* enhanced the colonization of *P. ananatis* by 1.99- to 7.96-fold across different time points and detection methods (Table 3).

**Table 3:** Population dynamics of *P. ananatis* in rice leaves and the inhibitory effect of *Xoo* co-inoculation.

Inoculation time (d)	*Pa* population in leaves (CFU/mL)			
	qPCR		Plate count	
	*Xoo+Pa*	*Pa*	*Xoo+Pa*	*Pa*
1	8.13 × 10^3^	4.08 × 10^3^	8.67 × 10^3^	4.00 × 10^3^
3	2.47 × 10^4^	6.07 × 10^3^	2.67 × 10^4^	5.67 × 10^3^
7	4.37 × 10^5^	7.94 × 10^4^	4.67 × 10^5^	8.67 × 10^4^
14	7.94 × 10^5^	1.46 × 10^5^	9.00 × 10^5^	1.13 × 10^5^

Pa stands for *P. ananatis*.

In this study, Pa (Lux) was used to evaluate the effect of *Xoo* inoculation on the population level of *P. ananatis* in rice leaves. Growth curve analysis revealed no significant difference in growth ability between Pa (Lux) and wild-type *P. ananatis* strain ZJU2 (Supplementary Figure S4B). The population size of *P. ananatis* was positively correlated with the bioluminescence intensity from the rice leaves. Over time, the bioluminescence intensity increased significantly in leaves co-inoculated with *Xoo* and *P. ananatis*, as well as in those inoculated with *P. ananatis* alone (Figure 9C). At each time point, the bioluminescence intensity was significantly higher in the co-inoculated leaves than in those inoculated with only *P. ananatis* (Figure 9D).

### 3.7 *Pantoea ananatis* alters the bacterial community structure in rice leaves infected with *Xoo*

**Figure 10:**
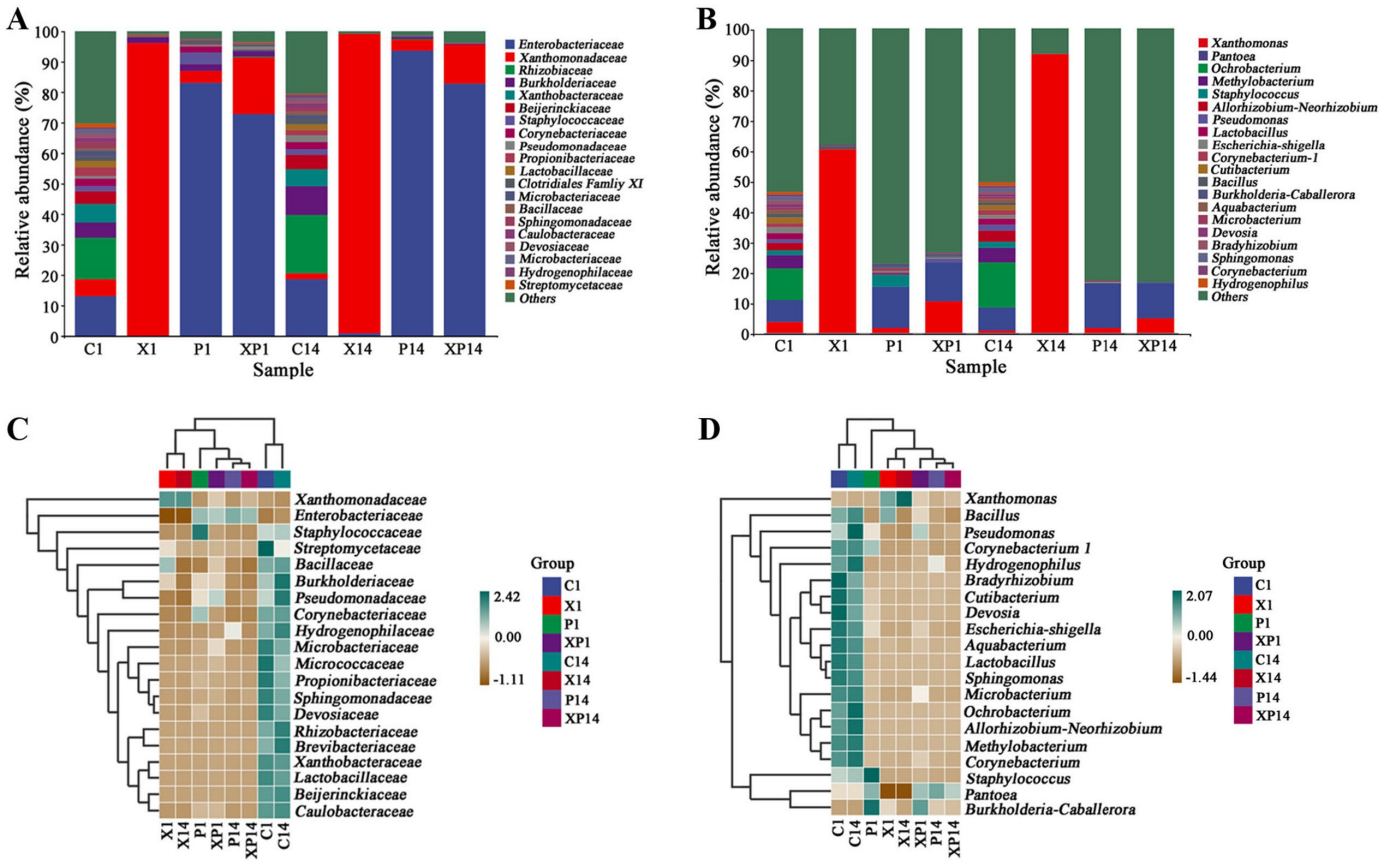
Bacterial community composition in rice leaves under different treatments. **(A)** Family-level species composition diagram. **(B)** Genus-level species composition diagram. **(C)** Family-level species composition heatmap. **(D)** Genus-level species composition heatmap. In the composition heatmap, samples and taxa (families/genera) were clustered via the Unweighted Pair-Group Method with Arithmetic Mean (UPGMA) based on Euclidean distance and Pearson correlation, respectively. C1: 1 d after no bacterial inoculation. X1: 1 d after *Xoo* inoculation. P1: 1 d after *P. ananatis* inoculation. XP1: 1 d after co-inoculation with *Xoo* and *P. ananatis*. C14: 14 d after no bacterial inoculation. X14: 14 d after *Xoo* inoculation. P14: 14 d after *P. ananatis* inoculation. XP14: 14 d after co-inoculation with *Xoo* and *P. ananatis*.

In this study, we found that microbial community structure was significantly influenced by the inoculated strains. At the family level, *Enterobacteriaceae*, *Xanthomonadaceae*, and *Rhizobiaceae* were the predominant bacterial groups in uninoculated groups (C1 and C14). In the *Xoo*-inoculated groups (X1 and X14), *Xanthomonadaceae* rapidly became dominant while other bacterial groups significantly declined, thereby indicating the strong inhibitory effect of Xoo on the indigenous community. On the contrary, in the *P. ananatis*-inoculated groups (P1 and P14), *Enterobacteriaceae* becoming the absolute dominant group, suggesting its robust colonization ability in leaves. In the co-inoculated with *Xoo* and *P. ananatis* groups (XP1 and XP14), the abundance of *Xanthomonadaceae* was markedly lower than in the *Xoo*-only inoculated group, demonstrating the sustained inhibitory effect of *P. ananatis* on *Xoo* (Figure 10A). At the genus level, different bacterial communities were observed in C1 and C14, with dominant genera of *Pseudomonas*, *Corynebacterium*, and *Methylobacterium*. In the XP1 and XP14, the relative abundances of bacterial groups like *Pantoea* and *Pseudomonas* recovered to near-healthy levels (Figure 10B). This indicated that *P. ananatis* can alleviate the microbial dysbiosis caused by *Xoo* infection and maintain the stability of the leaf micro-ecosystem.

Heatmap analysis further corroborated the results from the community composition analysis. In the X1 and X14, the abundance of *Enterobacteriaceae* and *Rhizobiaceae* decreased, suggesting that *Xoo* infection might suppress the growth of certain bacterial groups and lead to community structure imbalance. In the XP1 and XP14, *Enterobacteriaceae*, *Staphylococcaceae*, and *Burkholderiaceae* had higher abundances, with *Enterobacteriaceae* being dominant (Figure 10C). At the genus level, the relative abundance of *Xanthomonas* significantly increased in the X1 and X14, while the relative abundance of other genera significantly decreased. Compared to the X1 and X14, the relative abundance of *Xanthomonas* in the XP1 and XP14 significantly dropped (Figure 10D).

### 3.8 *Pantoea ananatis* alters the bacterial community diversity in rice leaves infected with *Xoo*

**Figure 11:**
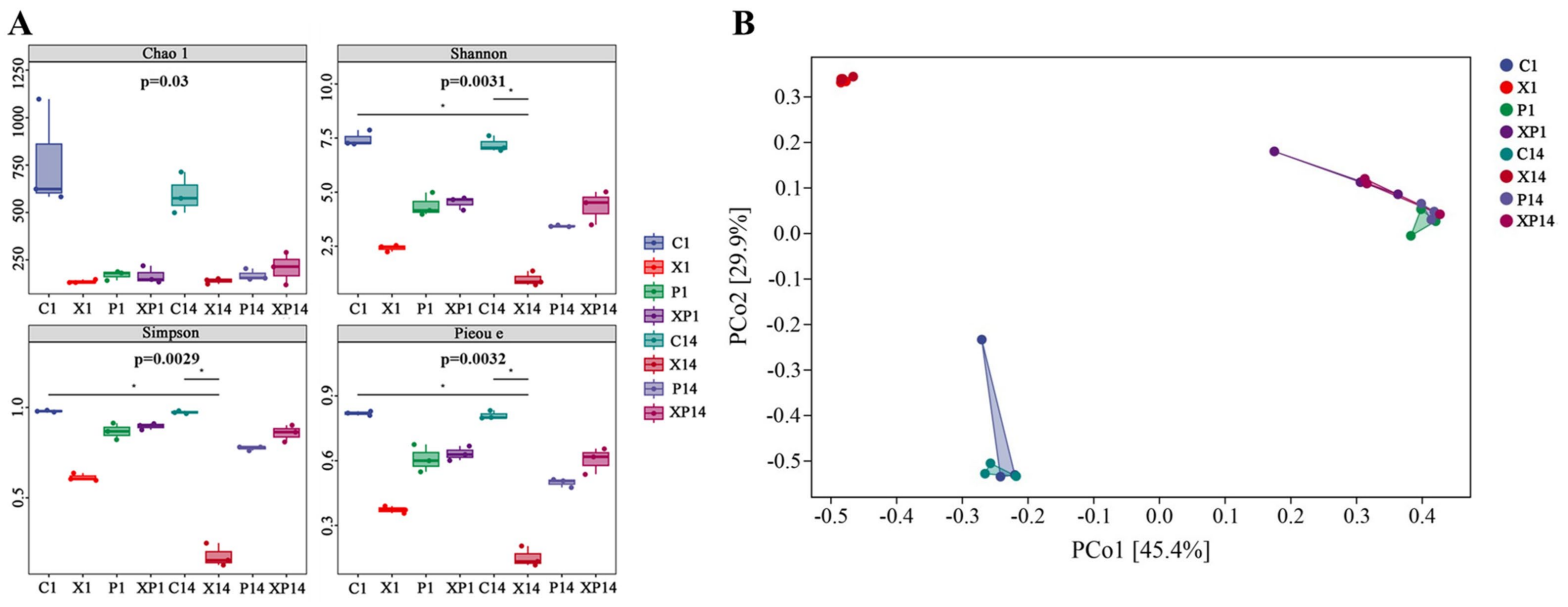
Alpha diversity and PCoA of bacterial communities in rice leaves under different treatments. **(A)** Box plots of alpha diversity indices (Chao1, Shannon, Simpson, and Pielou’s evenness). Different panels represent each index. The Kruskal-Wallis test *p*-value is shown for each index; pairwise significance (Dunns’ test) is marked above plots. **(B)** PCoA based on Bray-Curtis distances. Percentages in parentheses indicate the proportion of variance explained by each axis. C1: 1 d after no bacterial inoculation. X1: 1 d after *Xoo* inoculation. P1: 1 d after *P. ananatis* inoculation. XP1: 1 d after co-inoculation with *Xoo* and *P. ananatis*. C14: 14 d after no bacterial inoculation. X14: 14 d after *Xoo* inoculation. P14: 14 d after *P. ananatis* inoculation. XP14: 14 d after co-inoculation with *Xoo* and *P. ananatis*.

Differential bacterial treatments significantly impacted the alpha diversity of bacterial communities in rice leaves. Analysis of Chao1, Shannon, Simpson, and Pielou indices showed that C1 and C14 had the highest species richness and diversity, reflecting the natural abundance and balance of the leaf microbial community (Figure 11A). In contrast, X1 and X14 exhibited a significant decrease across all diversity indices. Specifically, X1’s Chao1, Shannon, Simpson, and Pielou indices decreased by 82.31, 67.60, 37.76, and 54.88%, respectively, compared to C1. Similarly, X14’s Chao1, Shannon, Simpson, and Pielou indices dropped by 76.53, 86.39, 81.44, and 81.48% compared to C14. However, in the XP1 and XP14, all diversity indicators were notably higher than in X1 and X14. For instance, XP1’s Shannon and Simpson indices increased by 86.88 and 47.54%, respectively, compared to X1. Furthermore, XP14’s Shannon and Simpson indices showed even more substantial increases of 378.57 and 377.78%, respectively, compared to X14.

Principal coordinate analysis (PCoA) revealed a clear separation among the sample groups, with the two principal coordinates (PCo1 and PCo2) explaining 45.4 and 29.9% of the total variation, respectively (Figure 11B). Community similarity between the uninoculated control groups (C1 and C14) and all inoculated groups (X1, P1, XP1, X14, P14, and XP14) was low, ranging from 1.34 to 8.52%. This demonstrated that bacterial inoculation significantly altered the resident leaf bacterial community structure. The temporal similarity within treatment groups was 62.15% for the *Xoo*-inoculated group (X1 vs. X14), 14.76% for the *P. ananatis*-inoculated group (P1 vs. P14), and 68.15% for the *P. ananatis* and *Xoo* co-inoculated group (XP1 vs. XP14). These results indicated that despite temporal shifts, the community composition within each inoculation group maintained a higher degree of consistency compared to the low similarity observed between different treatments. Besides, the community structure of *Xoo* and *P. ananatis* co-inoculated leaves more closely resembled that of leaves inoculated solely with *P. ananatis* rather than with *Xoo*. Specifically, the similarity between the P1 and XP1 communities was 68.64%, while that between the X1 and XP1 communities was only 18.33%. This disparity became even more pronounced by 14 dpi (75.29% vs. 9.53%).

## 4 Discussion

Rice bacterial leaf blight poses a serious threat to rice production. Although *Xoo* is widely recognized as the primary pathogen of rice bacterial leaf blight, recent studies have revealed that some *P. ananatis* strains not only act as companion pathogens causing similar symptoms but also exhibit biocontrol effects against this disease (Korinsak et al., 2021; Aksoy et al., 2023; Han et al., 2024; Yuan et al., 2024; Jiang et al., 2025). Therefore, this study conducted systematic identification and functional analysis of *P. ananatis* strains ZJU1-ZJU18 isolated from various regions in China, aiming to clarify their actual role in rice-microbe interactions. The findings not only provided key evidence to resolve the academic controversy of whether *P. ananatis* acts as a pathogen or a biocontrol agent but also revealed a novel and highly efficient indirect biocontrol mechanism that does not rely on direct antagonism.

Pathogenicity assays revealed that *P. ananatis* strains ZJU1-ZJU18 did not induce leaf blight symptoms under the tested conditions. While *P. ananatis* has been reported to cause various symptoms such as rice palea browning (Yan et al., 2010), the ability of strains ZJU1-ZJU18 to induce palea browning was not evaluated in this study. Whether these strains possess the potential to infect rice tissues during the reproductive stage and trigger grain diseases remains an independent and important scientific question, warranting dedicated future studies on rice during its reproductive growth phase.

Genomic analysis indicated that *P. ananatis* strain ZJU2 carries various virulence-associated factors (e.g., flagella and fimbriae biosynthesis proteins, non-fimbrial adhesins, amylovoran/stewartan-like exopolysaccharides, and pectinolytic enzymes) and mobile genetic elements. Previous studies have shown that these characteristics can facilitate horizontal gene transfer, introducing *P. ananatis* strain ZJU2 may induce leaf blight symptoms under field conditions (Stice et al., 2018; Yuan et al., 2023). Therefore, before developing these strains as biocontrol agents and deploying these in the field, systematic and long-term risk assessments must be conducted. These should encompass evaluations of its environmental adaptability and genetic stability, as well as indispensable steps such as small-scale field trials, tracking of its environmental fate, and assessments of its impacts on non-target organisms, including other microorganisms and beneficial insects. The plasmids utilized in this study lacked the essential genes for active conjugation, making horizontal gene transfer via this mechanism highly unlikely without helper plasmids. Thus, the risk of resistance gene dissemination to the phyllosphere microbiota under our experimental conditions is considered low. Furthermore, the expression of both antibiotic resistance and fluorescent markers likely carries a metabolic burden. The associated fitness cost may be more significant in the resource-limited phyllosphere or under microbial competition than was captured by our *in vitro* assays. This limitation highlights a key consideration and defines a clear objective for future work designed to more closely reflect practical environmental conditions.

The beneficial functions of PGP bacteria are attributed to their phosphate-solubilizing ability, siderophore production, and IAA synthesis, which facilitate the acquisition of essential nutrients required for plant growth (Krishnappa et al., 2025). *P. ananatis* strains ZJU1-ZJU18 exhibited multiple PGP traits. Although these traits were not directly linked to disease resistance in this study, they likely enhance host resistance indirectly by promoting plant growth, thereby increasing the potential of *P. ananatis* as a multifunctional biocontrol agent.

Results from greenhouse experiments involving the co-inoculation of *P. ananatis* and *Xoo* on rice demonstrated that *P. ananatis* exerted a significant control effect against rice bacterial leaf blight caused by *Xoo*. However, greenhouse experiments represent merely the first step in screening biocontrol strains, and the high efficacy observed in such trials requires subsequent validation under the more complex microecological conditions of field environments. Double-layer plate test revealed that neither the cells nor the metabolites of these strains produced inhibition zones against *Xoo*, indicating that their biocontrol mechanism does not rely on the secretion of diffusible antimicrobial compounds. Therefore, the research focus has shifted toward other non-antibacterial mechanisms, such as ecological interference or resource competition (Andongma et al., 2022; Ortiz et al., 2021).

Additionally, this study has not completely excluded the possibility of other non-diffusible mechanisms by *P. ananatis*, such as direct contact-dependent inhibition or interference with *Xoo* pathogenicity through quorum sensing disruption. Although frequent cell-to-cell contact occurs in the liquid co-culture system, the current experimental design cannot distinguish the relative contributions of contact inhibition and nutrient competition. Furthermore, *P. ananatis* may suppress *Xoo* by modifying the microenvironment, such as local pH or oxygen concentration. This plausible avenue of interference warrants further study.

Previous studies have shown that beneficial bacteria can inhibit pathogen growth through mechanisms such as the secretion of antibacterial compounds, niche competition, and nutrient competition (Shyntum et al., 2019; Wang L. et al., 2023; Leung et al., 2024; Wang X. et al., 2023; Cheng et al., 2025; Romão et al., 2025). This study observed that *P. ananatis* exhibited a faster growth rate than *Xoo* under pure culture condition. Flow cytometry analysis further confirmed that, even when the initial inoculum of *Xoo* was 100 times higher than that of *P. ananatis*, *P. ananatis* still accounted for over 90.53% of the total population in the co-culture system. Besides, *P. ananatis* maintained an absolute growth advantage (> 98.82%) in NL and CL broth. Nutritional competition might be the key factor for *P. ananatis* to inhibit the growth of *Xoo*. However, the exact mechanism requires further research.

Plate counting and qPCR analyses consistently demonstrated that *P. ananatis* significantly inhibited the colonization of *Xoo* on rice leaves. Compared to leaves inoculated with *Xoo*, those co-inoculated with *Xoo* and *P. ananatis* exhibited a reduction in the *Xoo* population density of over 96.78%. However, under co-inoculation conditions, there was no order-of-magnitude difference between *Xoo* and *P. ananatis*. This suggests that the *in vivo* advantage of *P. ananatis* may not stem solely from a higher absolute growth rate. We speculate that *Xoo* infection might inadvertently create a microenvironment that favors the efficient colonization of *P. ananatis* at leaf wound sites, thereby suppressing the colonization and expansion of *Xoo*.

Interestingly, this study found that the presence of *Xoo* significantly enhanced the colonization of *P. ananatis* in rice leaves. Based on the established paradigm in plant pathology, the suppression of host basal immunity (PTI) through strategies such as effector secretion is a common evolutionary strategy employed by pathogens to facilitate successful infection and may alter the microenvironment to favor the colonization of other microbes (Xu et al., 2024; Jiang et al., 2025). Therefore, we proposed that *Xoo* likely promoted the colonization of *P. ananatis* through multiple interconnected pathways. On the one hand, *Xoo* may secrete effectors to suppress rice PTI, thereby weakening the plant’s defense against subsequent invasion by *P. ananatis* and indirectly facilitating its colonization. On the other hand, the leaf cell damage caused by *Xoo* infection could lead to the leakage of nutrients such as sugars and amino acids, providing an additional nutritional source that supports the proliferation of *P. ananatis* (Jiang et al., 2025). These *Xoo*-induced modifications to host physiology and the leaf apoplastic environment are likely key to its facilitative effect on *P. ananatis*.

The qPCR method employed in this study targets a universal gene of *P. ananatis*. Therefore, it may theoretically detect both the inoculated Pa (Gen) strain carrying Gen resistance and naturally occurring endogenous *P. ananatis* populations in rice leaves. However, by incorporating subsequent colony verification using the specific genetic marker of the Pa (Gen) strain, we were able to minimize the likelihood of misattributing endogenous populations to the inoculated strain. Our study revealed that the population of *P. ananatis* in leaves co-inoculated with *Xoo* and *P. ananatis* was 1.99 to 7.96 times higher than that in leaves inoculated solely with *P. ananatis*. We speculated that *Xoo* infection may have altered the leaf microenvironment or triggered plant physiological responses, thereby creating more favorable conditions for the proliferation of *P. ananatis*. The unique interaction between *Xoo* and *P. ananatis* not only highlights the complexity of microbial interaction processes but also provides novel insights for developing disease control strategies based on the targeted amplification of beneficial bacterial species.

The structural dynamics of the leaf microbial community have direct or indirect effects on disease development. Community composition analysis and heatmap results demonstrated that *P. ananatis* can inhibit *Xoo* proliferation in leaves, alleviate the microbial dysbiosis caused by *Xoo* infection and maintain the stability of the leaf micro-ecosystem. Alpha diversity analysis results confirmed that *Xoo* infection markedly reduces the richness and diversity of the leaf microbial community, whereas the introduction of *P. ananatis* effectively mitigates this decline, helping to change community structure and guide a shift towards a healthier microbial state. PCoA results indicated that *P. ananatis* exerts a stronger influence than *Xoo* in modulating the structure of the leaf microbial community. We thought that *P. ananatis* may preferentially colonize and compete for nutrients to occupy ecological niches, thereby creating a microecological environment unfavorable for *Xoo* survival, so as to modulate the composition and function of the microbial community, and ultimately achieving sustained and stable biocontrol effects. In future studies, integrating tools such as PICRUSt2 for functional prediction and employing techniques like meta-transcriptomics to validate causal relationships will help position the findings of this research as an initial step for subsequent mechanistic investigations.

## 5 Conclusion

In conclusion, this study successfully isolated and identified *P. ananatis* strains ZJU1-ZJU18 and systematically evaluated their biocontrol potential against rice bacterial leaf blight. *P. ananatis* strains ZJU1-ZJU18 did not induce leaf blight symptoms under the tested conditions and possessed significant PGP potential. Furthermore, their co-inoculation with *Xoo* significantly suppressed the development of rice bacterial leaf blight. *P. ananatis* maintains a sustained growth advantage in culture medium, which may be attributed to its strong nutrient competitiveness. Co-inoculation of *P. ananatis* with *Xoo* resulted in a significant reduction in the population of *Xoo* in the leaves, while enhancing the colonization of *P. ananatis*. 16S amplicon sequencing analysis showed that *Xoo* infection disrupted the homeostasis of the leaf microbiome, significantly reducing its diversity. Inoculation with *P. ananatis* can reduce the relative abundance of *Xoo* in rice leaves. *P. ananatis* exerts a stronger effect than *Xoo* in modulating the structure of the leaf microbial community. Our results supported that nutrient competition is a key factor in the inhibition of *Xoo* by *P. ananatis* and preliminarily reveals its suppressive effect at the community level, providing a new theoretical foundation and microbial resource for the green and sustainable management of rice bacterial leaf blight.

## Data Availability

The sequence information used in this study can be accessed via the Genbank database (https://www.ncbi.nlm.nih.gov/genbank) using the accession numbers given in Data Sheet 1.

## Footnotes

1
https://www.ezbiocloud.net/tools/ani

2
https://www.genescloud.cn/login
