# Supplementary material for: Mechanism of Pantoea ananatis in the biocontrol of rice bacterial leaf blight
Source: Front Microbiol. 2026 Feb 4;17:1722838. doi: 10.3389/fmicb.2026.1722838 (PMC12913557; doi:10.3389/fmicb.2026.1722838)
Supplement: Supplementary file 1 [file Data_Sheet_1.docx]

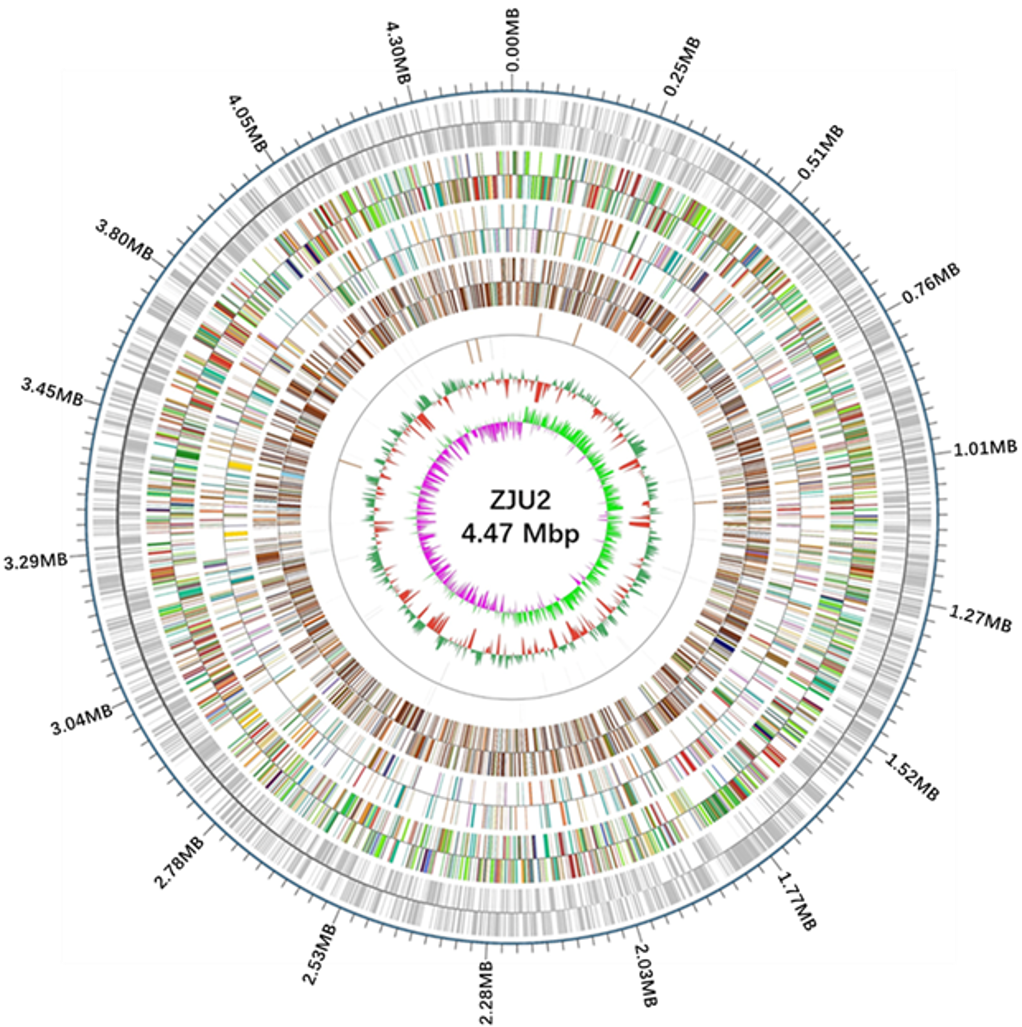


**Figure S1. Genomic circle map of strain ZJU2.** From the outside to the inside, the genome circular plot consists of the following components: genomic sequence position coordinates, protein-coding genes, gene functional annotation results, ncRNAs, genomic GC content, and genomic GC skew values. The GC content is calculated using a sliding window of (chromosome length/1000) bp with a step size of (chromosome length/1000) bp. Red inward peaks indicate regions where the GC content is lower than the genome-wide average, while green outward peaks indicate regions where it is higher. The height of a peak corresponds to the magnitude of deviation from the average GC content. GC skew was computed with a sliding window of (chromosome length/1000) bp and a step size of (chromosome length/1000) bp, using the formula (G – C)/(G + C). Pink inward peaks denote regions where G content is lower than C content, while light green outward peaks indicate the opposite.


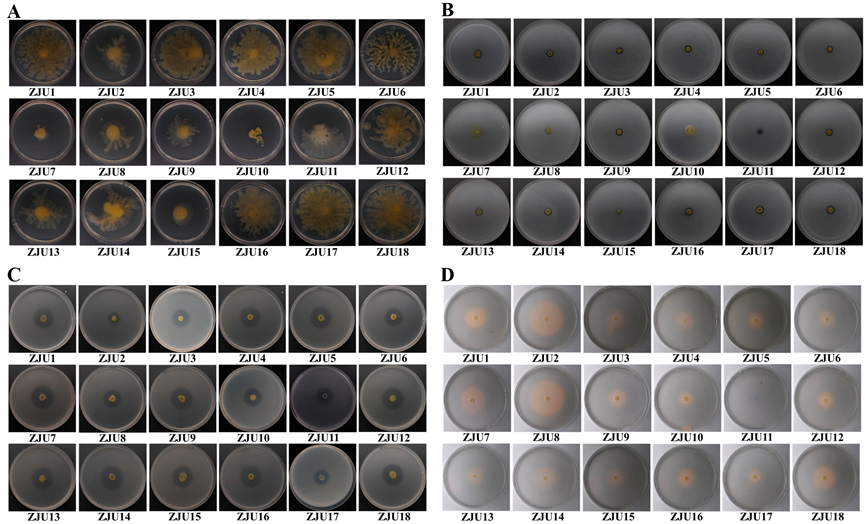


**Figure S2. Promoting properties of *P. ananatis* strain ZJU1-ZJU18.** (A): Motility. (B): Inorganic phosphorus solubilization. (C): Organic phosphorus solubilization. (D): Siderophore production. . .
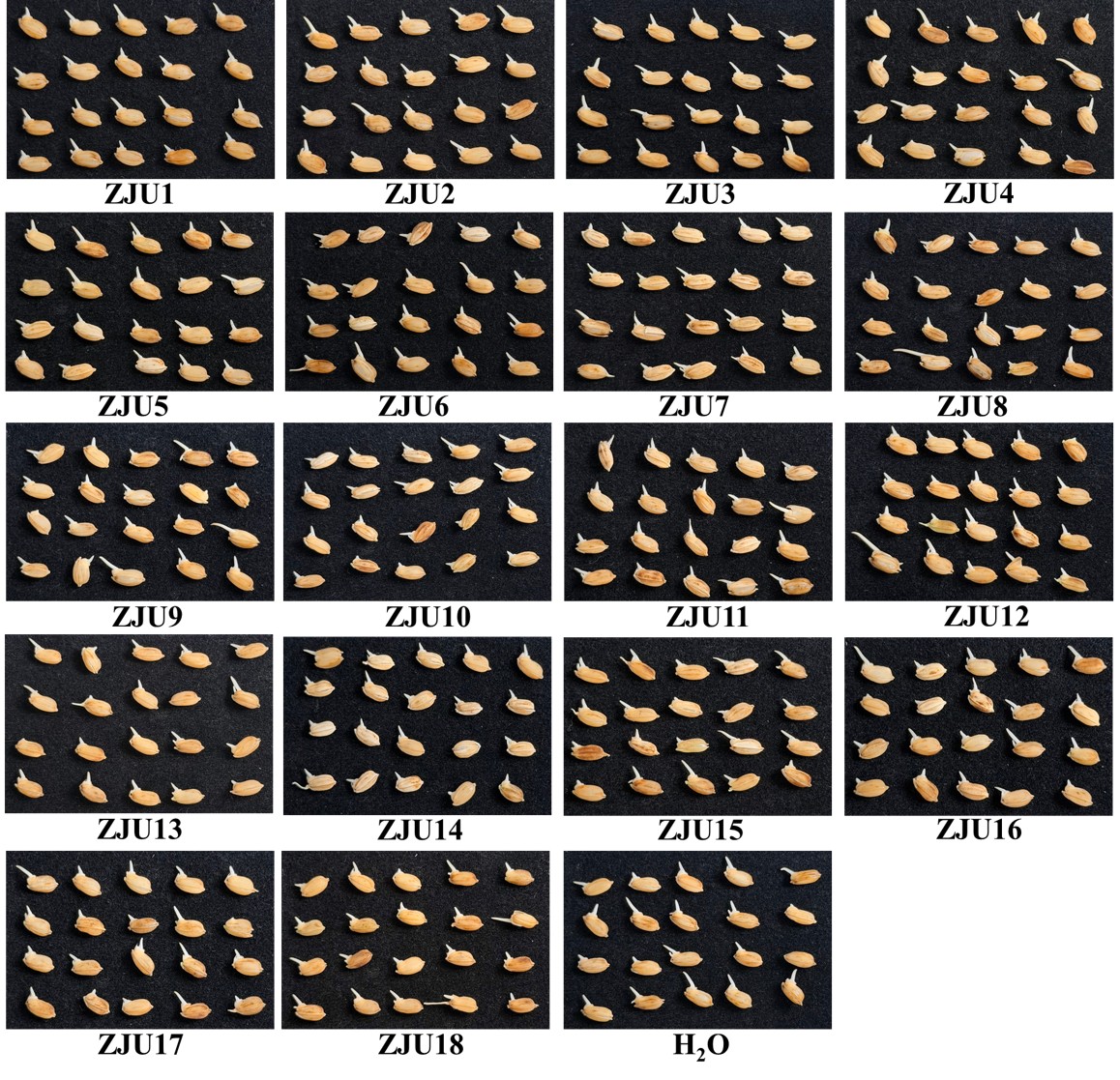


**Figure S3.** **Effect of *P. ananatis* on rice seed germination.**


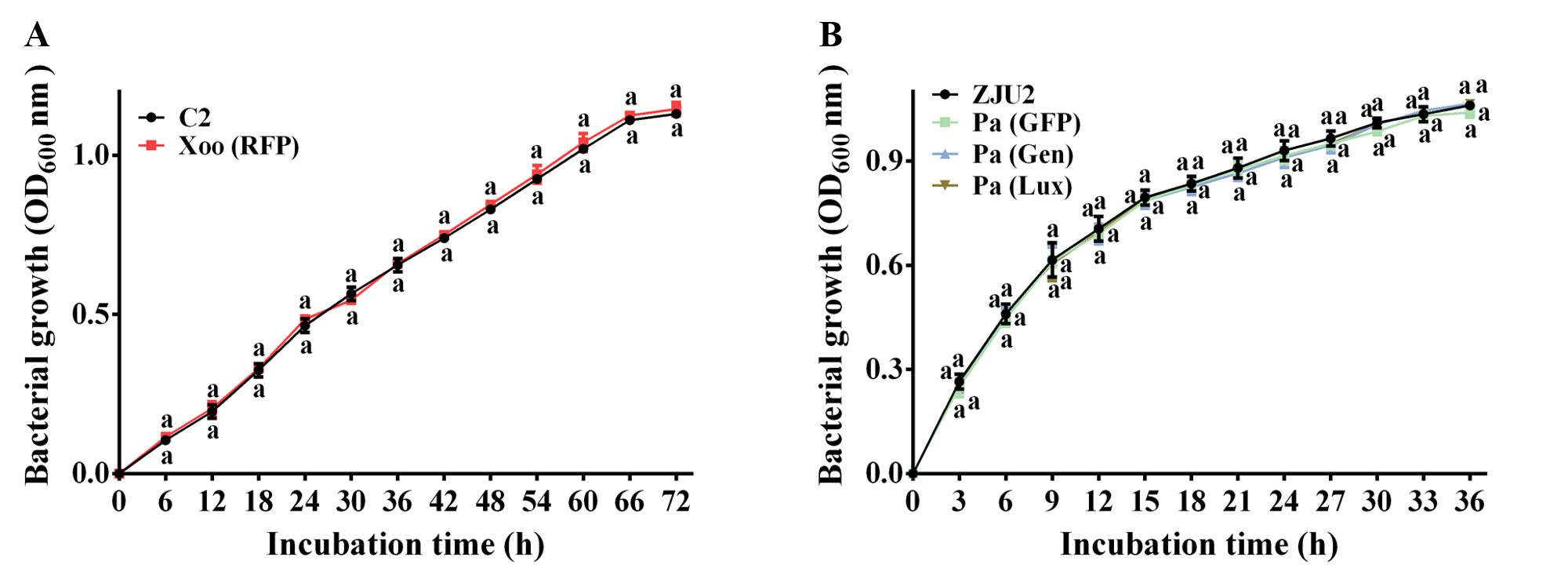


**Figure S4. Determination of bacterial growth curve.** (A): *Xoo*. (B): *P. ananatis.* Pa represents *P. ananatis.* C2 is the wild-type strain of *Xoo*, ZJU2 is the wild-type strain of *P. ananatis*. Statistical differences were analyzed by one-way ANOVA. Different lowercase letters within the same time point and assay indicate significant differences between treatments (*P* < 0.05).


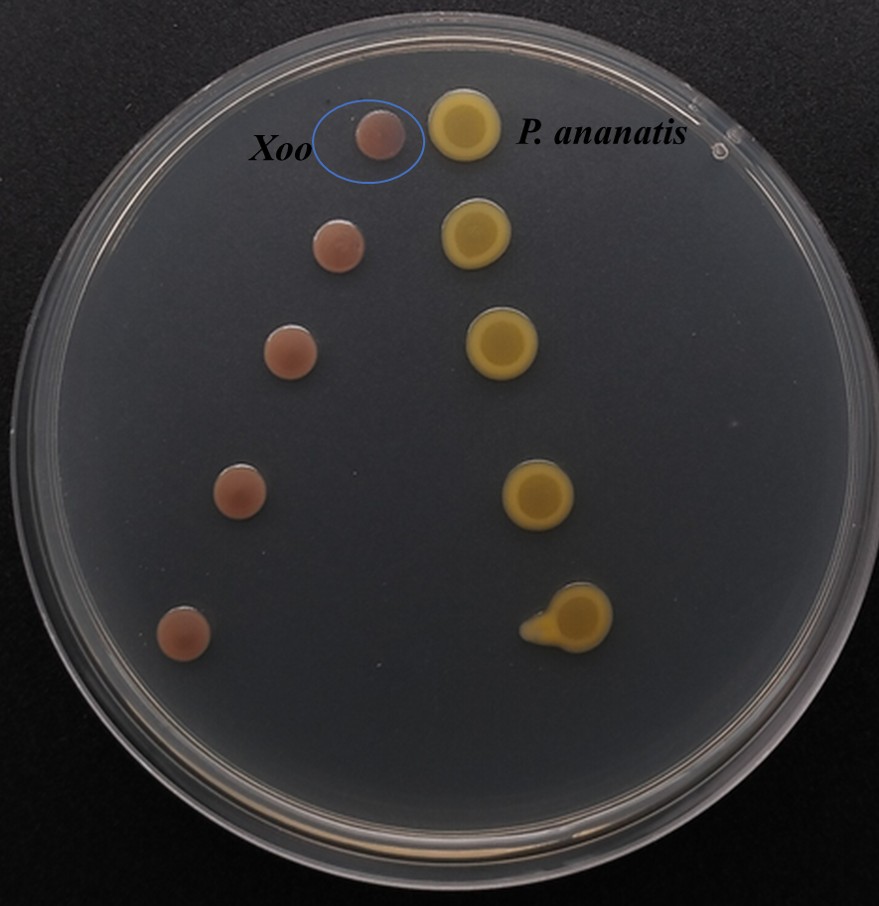


**Figure S5. Spot assay of *Xoo* and *P. ananatis.***

**Table S1. The primers used in this study.**

| **Name** | **Nucleotide sequence (5'–3')** | **Application** |
| --- | --- | --- |
| 16S-F | AGAGTTTGATCCTGGCTCAG | Bacterial identification |
| 16S-R | GGTTACCTTGTTACGACTT |  |
| AatpD-F | RTAATYGGMGCSGTRGTNGAYGT | *AtpD* amplification |
| AatpD-R | TCATCCGCMGGWACRTAWAYNGCCTG |  |
| AgyrB-F | TAARTTYGAYGAYAACTC YTAYAAAGT | *GyrB* amplification |
| AgyrB-R | CMCCYTCCACCARGTAMAGTT |  |
| AfusA-F | CATCGGTATCAGTGCKCACATCGA | *fusA* amplification |
| AfusA-R | CAGCATCGCCTGAACRCCTTTGTT |  |
| ArpoB -F | AACCAGTTCCGCGTTGGCCTG | *RpoB* amplification |
| ArpoB-R | CCTGAACAACACGCTCGGA |  |
| SatpD-F | TGCTGGAAGTKCAGCARCAG | *AtpD* sequencing |
| SatpD-R | CCMAGYARTGCGGATACTTC |  |
| SgyrB -F | GTVCGTTTCTGGCCVAG | *GyrB* sequencing |
| SgyrB-R | CTTTACGRCGKGTCATWTCAC |  |
| SfusA-F | CATCGGTATCAGTGCKCACATCGA | *FusA* sequencing |
| SfusA-R | CAGCATCGCCTGAACRCCTTTGTT |  |
| SrpoB-1F | CAGTTCCGCGTTGGCCTG | *RpoB* sequencing |
| SrpoB-2F | TGATCAACGCCAAGCC |  |
| SrpoB-R | CGGACCGGCCTGACGTTGCAT |  |
| qXoo-F | GCCGCTAGGAATGAGCAAT | Quantitative *Xoo* |
| qXoo-R | GCGTCCTCGTCTAAGCGATA |  |
| qPa-F | GTATCCGCGCCTTTGTTGAGT | Quantitative *P. ananatis* |
| qPa-R | AATGCCGTCTTTCTCGGTTGA |  |

**Table S2. Accession numbers of the *atpD*, *fusA*, *gyrB*, and *rpoB* genes for *P. ananatis* strains ZJU1 to ZJU18.**

| **Strains** | **NCBI accession number of conserved genes** | | | |
| --- | --- | --- | --- | --- |
|  | *atpD* | *fusA* | *gyrB* | *rpoB* |
| ZJU1 | PX776223 | PX776241 | PX776259 | PX776277 |
| ZJU2 | PX776224 | PX776242 | PX776260 | PX776278 |
| ZJU3 | PX776225 | PX776243 | PX776261 | PX776279 |
| ZJU4 | PX776226 | PX776244 | PX776262 | PX776280 |
| ZJU5 | PX776227 | PX776245 | PX776263 | PX776281 |
| ZJU6 | PX776228 | PX776246 | PX776264 | PX776282 |
| ZJU7 | PX776229 | PX776247 | PX776265 | PX776283 |
| ZJU8 | PX776230 | PX776248 | PX776266 | PX776284 |
| ZJU9 | PX776231 | PX776249 | PX776267 | PX776285 |
| ZJU10 | PX776232 | PX776250 | PX776268 | PX776286 |
| ZJU11 | PX776233 | PX776251 | PX776269 | PX776287 |
| ZJU12 | PX776234 | PX776252 | PX776270 | PX776288 |
| ZJU13 | PX776235 | PX776253 | PX776271 | PX776289 |
| ZJU14 | PX776236 | PX776254 | PX776272 | PX776290 |
| ZJU15 | PX776237 | PX776255 | PX776273 | PX776291 |
| ZJU16 | PX776238 | PX776256 | PX776274 | PX776292 |
| ZJU17 | PX776239 | PX776257 | PX776275 | PX776293 |
| ZJU18 | PX776240 | PX776258 | PX776276 | PX776294 |

**Table S3. Basic information of *Pantoea* strains.**

| **Strain** | **Accession number** | **Most closely related strain** | **Similarity** | **Source** |
| --- | --- | --- | --- | --- |
| ZJU1 | SUB14338872 | *Pantoea* sp. RSPAM1 | 99.72% | Wenzhou |
| ZJU2 | SUB14338955 | *P. ananatis* CU: ASL: N3 | 99.09% | Hangzhou |
| ZJU3 | SUB14343598 | *P. ananatis* SGAir0210 | 99.86% | Hangzhou |
| ZJU4 | SUB14343599 | *P. ananatis* SGAir0210 | 99.93% | Quzhou |
| ZJU5 | SUB14343602 | *P. ananatis* SGAir0210 | 100.00% | Shaoxing |
| ZJU6 | SUB14343612 | *P. ananatis* SGAir0210 | 100.00% | Ningbo |
| ZJU7 | SUB14343619 | *P. ananatis* NN08200 | 99.93% | Ningbo |
| ZJU8 | SUB14343644 | *P. ananatis* NN08200 | 99.93% | Taizhou |
| ZJU9 | SUB14343648 | *P. ananatis* SGAir0210 | 100.00% | taizhou |
| ZJU10 | SUB14343653 | *P. ananatis* SGAir0210 | 99.93% | Jinhua |
| ZJU11 | SUB14343661 | *P. ananatis* SGAir0210 | 99.93% | Suqian |
| ZJU12 | SUB14338960 | *P. ananatis* SGAir0210 | 99.86% | Huai'an |
| ZJU13 | SUB14338963 | *P. ananatis* CU: ASL: N3 | 99.72% | Huai'an |
| ZJU14 | SUB14338966 | *P. ananatis* SGAir0210 | 99.10% | Anqing |
| ZJU15 | SUB14343530 | *P. ananatis* OsEp_Plm_30B19 | 100.00% | Anqing |
| ZJU16 | SUB14343522 | *P. ananatis* SGAir0210 | 99.86% | Chizhou |
| ZJU17 | SUB14343516 | *P. ananatis* OsEp_Plm_30B19 | 100.00% | Guiping |
| ZJU18 | SUB14343513 | *Pantoea* sp. RSPAM1 | 99.79% | Jiangmen |

Table S4. Swimming diameter of *P. ananatis* strains ZJU1-ZJU18.

| **Strains** | **Swimming diameter (mm)** | **Strains** | **Swimming diameter (mm)** |
| --- | --- | --- | --- |
| ZJU1 | 61.67±1.53a | ZJU10 | 15.33±0.58i |
| ZJU2 | 41.10±0.94f | ZJU11 | 52.67±0.24d |
| ZJU3 | 53.17±1.03d | ZJU12 | 55.33±0.47c |
| ZJU4 | 60.67±1.25a | ZJU13 | 56.33±1.70c |
| ZJU5 | 58.33±0.85b | ZJU14 | 61.10±0.82a |
| ZJU6 | 48.33±1.53e | ZJU15 | 31.20±1.18g |
| ZJU7 | 17.67±0.58h | ZJU16 | 49.33±0.94e |
| ZJU8 | 41.07±1.01f | ZJU17 | 61.33±0.53a |
| ZJU9 | 41.67±1.53f | ZJU18 | 62.67±0.57a |

One-way analysis of variance was used to test the significance of differences between groups. Data marked with different lowercase letters indicated statistical differences between groups (*P <* 0.05).

**Table S5.** **Germination rate of rice seeds inoculated with *P. ananatis* strains ZJU1-ZJU18.**

| **Treatments** | **Germination rate (%)** | **Treatments** | **Germination rate (%)** |
| --- | --- | --- | --- |
| ZJU1 | 98.33±2.89a | ZJU11 | 96.67±2.89a |
| ZJU2 | 96.67±2.89a | ZJU12 | 96.67±5.77a |
| ZJU3 | 95.00±5.00a | ZJU13 | 93.33±2.89a |
| ZJU4 | 96.67±2.89a | ZJU14 | 95.00±0.00a |
| ZJU5 | 96.67±5.77a | ZJU15 | 98.33±2.89a |
| ZJU6 | 95.00±5.00a | ZJU16 | 96.67±5.77a |
| ZJU7 | 95.00±0.00a | ZJU17 | 95.00±0.00a |
| ZJU8 | 98.33±2.89a | ZJU18 | 95.00±5.00a |
| ZJU9 | 96.67±2.89a | PBS | 93.33±2.89a |
| ZJU10 | 96.67±2.89a |  |  |

PBS represents phosphate-buffered saline buffer*.* One-way analysis of variance was used to test the significance of differences between groups. Data marked with different lowercase letters indicated statistical differences between groups (*P <* 0.05).
